# Multifaceted Interactions of Thermally Activated Delayed Fluorescent Emitters with Dielectric Environments: Charge Transfer vs. Structural Relaxation

**DOI:** 10.3390/molecules31101581

**Published:** 2026-05-09

**Authors:** Yiran Tian, Yaxin Wang, Yixuan Gao, Zilong Guo, Shaowen Chu, Yonghang Li, Yandong Han, Wensheng Yang, Xiaonan Ma

**Affiliations:** 1 Institute of Molecular Plus, Tianjin University, Tianjin 300072, China; 2 Engineering Research Center for Nanomaterials, Henan University, Kaifeng 475004, China

**Keywords:** thermally activated delayed fluorescence, nonradiative decay rate, vibronic effects, charge transfer, structural relaxation

## Abstract

Thermally activated delayed fluorescence (TADF) emitters doped in host–guest systems are widely utilized for organic light-emitting diodes (OLEDs), where key rate constants and the fluorescence quantum yield (Φ_F_) are strongly influenced by the surrounding environment. However, the multifaceted interactions, i.e., dipole–dipole interaction and conformational restraint between the emitter and environment have been rarely investigated systematically, where excited state charge transfer (CT) and structural relaxation (SR) of emitters should be considered equally. In this study, four representative CT–TADF emitters were selected as model systems and studied in PS/PMMA:TADF:CA host–guest doped films with varied dielectric constants and matrix rigidity. Within D–A and D–A–D configurations, donor substitution from PXZ to DMAC varied CT characteristics, whereas TRZ-based D–A and DPS-based D–A–D emitters provided a relative difference in SR owing to their different rigidity. The total reorganization energy (*λ*_Total_) was introduced as a quantitative measure of these multifaceted interactions and correlated with the rate constants. The results indicate that the dielectric dependence of the nonradiative decay rate (*k*_nr_^S^) for D–A–D molecules cannot be explained by the simplified energy gap law, where the vibronic effect plays the role of a game changer. This work provides a quantitative framework and highlights vibrational frequency as a key design parameter for optimizing Φ_F_ in host–guest doped OLED devices.

## 1. Introduction

Organic light-emitting diodes (OLEDs) have emerged as a promising technology for next-generation displays, owing to their superior electroluminescence, flexibility, and tunable colors [1,2,3,4,5]. To suppress concentration-induced quenching and improve the stability of the emitting layer during fabrication and operation, host–guest doped films are widely employed in OLEDs [6,7,8,9]. In such systems, the fluorescence properties of emitters are determined not only by their intrinsic molecular characteristics but also by interactions with the surrounding environment. These interactions encompass both electrostatic effects, such as dipole–dipole interactions arising from charge transfer (CT) characteristics, and the conformational constraint associated with the structural relaxation (SR) of emitters in the excited states [10,11,12,13,14,15], which we refer to as multifaceted interactions. Early studies on fluorescent materials, such as the red laser dye DCM2, primarily attributed host–guest interactions to electrostatic effects, which were clarified by incorporating polar dopants to alter the local electric field within the films [16,17]. However, the third-generation thermally activated delayed fluorescence (TADF) emitters, with spatially separated frontier orbitals exhibiting both CT and SR characteristics, display multifaceted interactions compared to conventional fluorophores [18,19,20,21,22], i.e., co-existence of dipole–dipole interactions and conformational constraint in host–guest doped films.

In previous investigations, ternary solid films, i.e., polystyrene (PS)/poly(methyl methacrylate) (PMMA): PXZ-PYR/ACRPyr: camphoric anhydride (CA), demonstrated that the spectral shifts in prompt and delayed fluorescence over time depended dominantly on the degree of conformational constraint, independent of dielectric constants [23]. This observation indicates that SR characteristics are integral to host–guest interactions and may partly change emission behavior [12,24,25,26,27]. In analogous ternary films (e.g., PS:2PXZ-OXD:CA), the fluorescence quantum yield (Φ_F_) exhibited a non-monotonic dependence on CA concentration [28]. This trend implies competition between multiple processes, such as nonradiative decay (*k*_nr_^S^, S_1_→S_0_) and reverse intersystem crossing (*k*_RISC_, T_1_→S_1_), which determine the contributions of prompt (Φ_PF_) and delayed fluorescence (Φ_DF_). A quantitative analysis of these rates is therefore essential to provide a perspective on the relationship between multifaceted interactions and emitting efficiency [29,30,31,32].

In general, both *k*_RISC_ and *k*_nr_^S^ are fundamentally governed by Fermi’s golden rule, in which the transition probability is jointly determined by the energy gap and electronic coupling between the initial and final states [33]. In most previous discussions, the environment-dependent Φ_F_ of TADF emitters has been primarily explained through changes in energy gaps, since dipole–dipole interaction can effectively stabilize the emitting CT excited states [34,35,36,37,38,39]. Nevertheless, we have recently demonstrated that the environment of doped films can also constrain molecular conformational flexibility and thereby alter the vibronic contribution to the key rate constants [40]. In rigid matrices, the structural relaxation of an ADA-type TADF emitter is significantly suppressed, leading to a marked reduction in *k*_nr_^S^ by weakening the contribution of the promoting vibrational modes. Thus, the distinctive TADF photophysics characterized by CT and SR characteristics, together with the host–guest multifaceted interactions, can affect energy gaps and vibronic contributions that govern key decay rates, thereby influencing the emission behavior.

In this work, rigid D–A and flexible D–A–D type CT–TADF emitters were employed to construct ternary PS/PMMA:TADF:CA host–guest doped films. By systematically varying dielectric constants and film rigidity and employing the total reorganization energy (*λ*_Total_) as quantitative indicator of multifaceted interactions, an integrated assessment of CT and SR characteristics was performed for the selected emitters in the doped films. On this basis, the key rate *k*_RISC_ and *k*_nr_^S^ are extracted and analyzed using steady and time-resolved spectroscopy with assistance of theoretical calculations. In particular, D–A–D type emitters exhibit an abnormal dependence of *k*_nr_^S^ dielectric constants of the doped films, revealing that energy gap and vibronic effects must be simultaneously considered. These results show that considering host–guest effects only in terms of the dielectric stabilization of CT states may lead to deviations in predicting photophysics, because external structural restraint and the associated vibronic contribution, especially to *k*_nr_^S^, must also be taken into account.

## 2. Results and Discussion

### 2.1. Emitting Properties

**Scheme 1:**
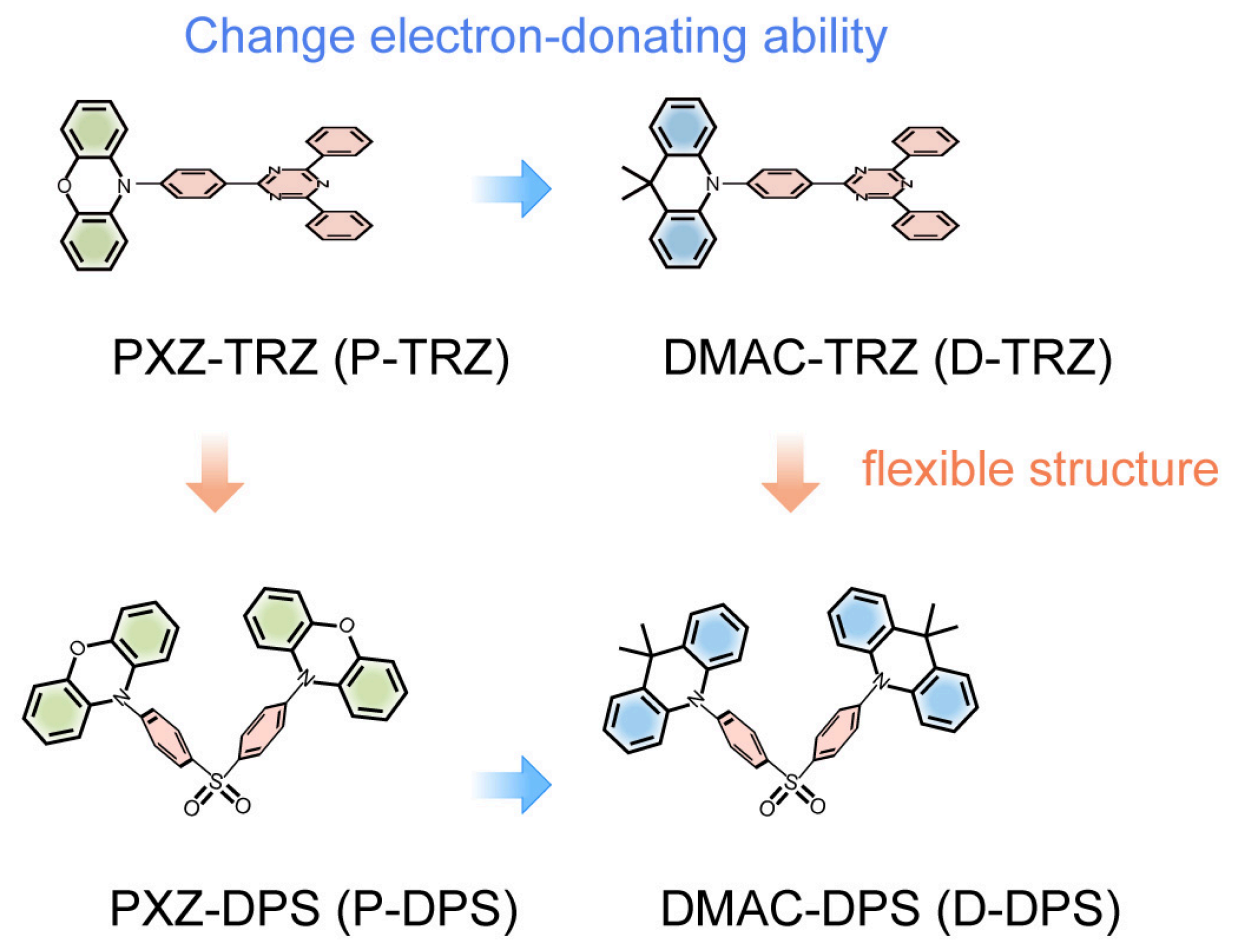
Molecular structures of the four TADF emitters studied in this work, illustrating the variation in donor electron-donating ability (PXZ vs. DMAC) and molecular flexibility (rigid D–A vs. flexible D–A–D structures).

To investigate the host–guest multifaceted interactions in doped films, two variables were introduced to the molecular structure of the TADF emitters, corresponding to SR and CT characteristics, respectively. Firstly, 2,4,6-triphenyl-1,3,5-triazine (TRZ) with a relatively planar geometry and diphenyl sulfone (DPS) with a highly twisted geometry were chosen as electron acceptors, corresponding to the more rigid TRZ-based D–A and more flexible DPS-based D–A–D emitters, respectively [41,42,43]. For the D–A structure, phenoxazine (PXZ, abbreviated as P) and 9,9-dimethyl-9,10-dihydroacridine (DMAC, abbreviated as D) were introduced as donors, leading to changed CT characteristics (emitters P-TRZ and D-TRZ). The corresponding D–A–D emitters P-DPS and D-DPS were similarly included. The four emitters investigated in this work were commercially obtained and were not newly synthesized in the present study. In the investigated D–A emitters, the electron donors exhibit nearly identical orthogonal dihedral angles with the TRZ acceptor, leading to a rigid structure and minimized differences in SR characteristics. Similarly, the more flexible D–A–D type emitters were constructed with PXZ and DMAC donors incorporated with the DPS acceptor, denoted as P-DPS and D-DPS, respectively [44,45,46,47,48]. The chemical structures of the involved TADF emitters are shown in Scheme 1.

In addition to varying the CT and SR characteristics of the TADF emitters, two environmental factors were also tuned: (i) the dielectric constants within the doped films, controlled by the CA concentration. The dielectric constants of the matrices were tuned by varying the CA concentration, and estimated using published linear relationships [23], and (ii) the environment rigidity experienced by the emitters (PS and PMMA), where PS was reported to provide a stronger conformational restraint for guest emitters than PMMA [23,49]. All controlled variables are summarized in Table 1.

Quantum chemical calculations were performed to clarify the intrinsic excited-state characteristics of the involved emitters. The ground (S_0_) and excited-state (S_1_, T_1_) geometries were optimized at the PBE0/6-311g** level using the PCM solvation model with toluene (*ε* = 2.37). PBE0 was selected from six widely used hybrid and long-range corrected functionals because, with its 25% HF exchange, it provides a reasonable balance between the electronic-structure features of CT emitters and computational consistency for the subsequent geometry, charge-transfer, and vibronic analyses, while remaining consistent with the experimental fluorescence energies in toluene solution (Table S1 and Figure S1) [50]. PCM was used to account for the bulk dielectric response of the surrounding medium, as the excited-state properties of molecules in the environment are strongly affected by polarization [51]. As a continuum solvation model, PCM does not account for conformational constraint. Thus, if only the dielectric effect is considered, while neglecting the conformational restriction imposed by PS, toluene is a reasonable approximation, owing to its dielectric constant being similar to that of PS. The calculated *E*(S_1_) values are in reasonable agreement with the fluorescence energies measured in PS films and toluene solution (Table S2).

The CT characteristics of S_1_ states were evaluated using Multiwfn [52,53], while the relevant parameters and physical meanings are illustrated in Table S3. All involved emitters exhibited typical CT characteristics with a CT% of the S_1_ state up to 91% for D–A and 87% for D–A–D emitters, indicating that the local excited (LE) contribution to S_1_ remains limited. However, further analysis revealed clear differences between the D–A and D–A–D emitters. The *D* index, quantifying the spatial distance between the electron and hole centroids [54], was considerably larger for the D–A (P-TRZ: 6.17 Å; D-TRZ: 6.03 Å) than for the D–A–D emitters (P-DPS: 2.53 Å; D-DPS: 2.46 Å). Likewise, the *t* index of the D–A emitters (P-TRZ: 4.22; D-TRZ: 4.05), which quantifies the degree of charge separation along the CT direction [54], was observed to be significantly higher than that of the corresponding D–A–D counterparts (P-DPS: 1.00; D-DPS: 0.90). These quantitative charge transfer parameters are further supported by the vertical excitation analysis and the frontier molecular orbital distributions (Tables S4 and S5). As shown in Table S4, the S_1_ states of the D–A emitters are dominated almost exclusively by the HOMO→LUMO transition, whereas the D–A–D emitters exhibit slightly more mixed transition compositions involving additional contributions from adjacent frontier orbitals. Accordingly, Table S5 shows that the HOMOs of the D–A emitters are mainly localized on the donor moieties, while the LUMOs are concentrated on the acceptor units, leading to a more pronounced spatial separation between the hole and electron. By contrast, for the D–A–D emitters, the donor units are located on both sides of the acceptor, so the hole centroid is determined by the vector sum of the two donor contributions, resulting in a smaller hole–electron separation and thus a smaller effective *D* and *t* index. These results clearly indicate more evident CT characteristics in D–A than in the corresponding D–A–D emitters.

**Figure 1:**
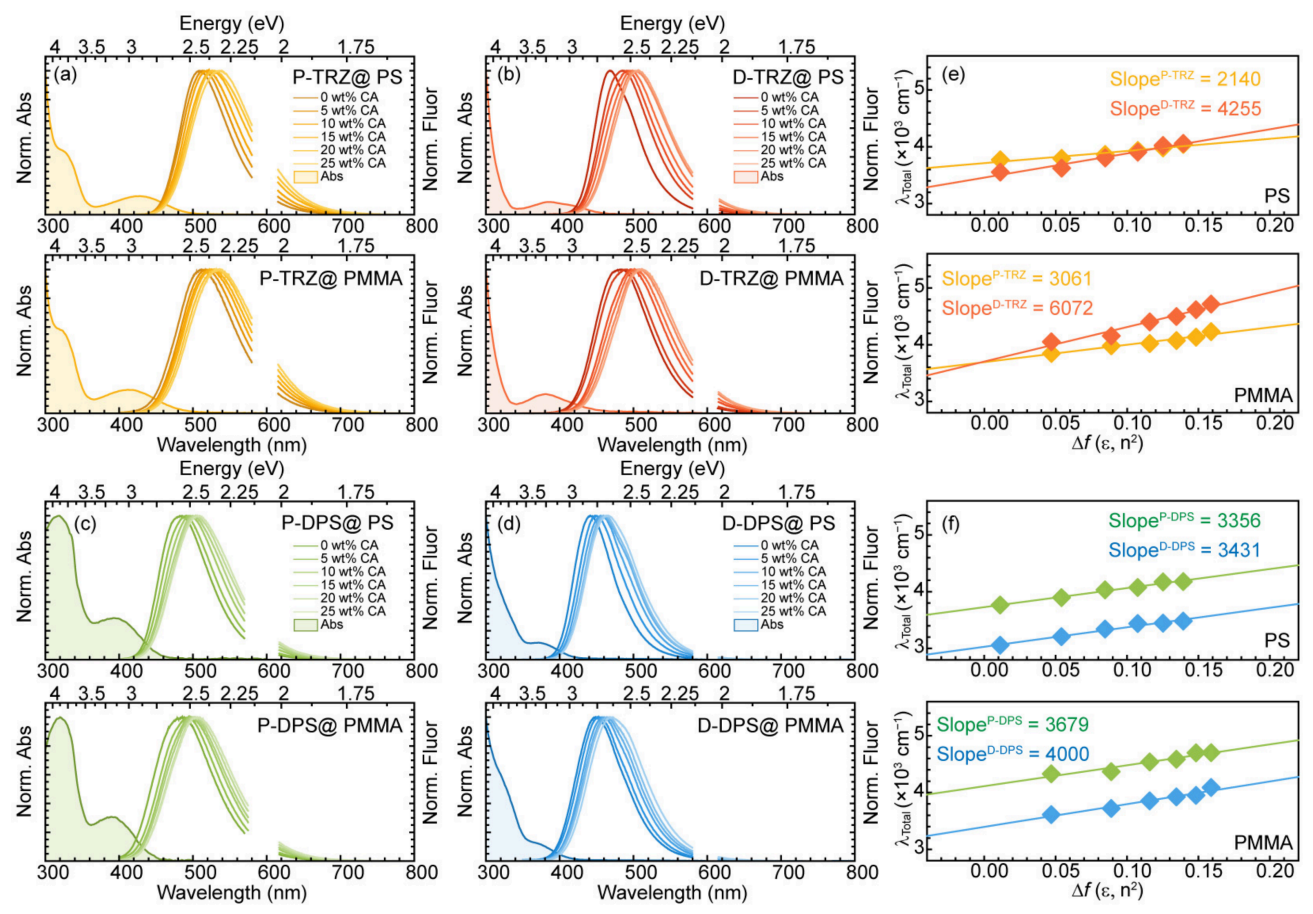
Normalized steady-state absorption and fluorescence spectra recorded under 300 nm of P-TRZ (**a**), D-TRZ (**b**), P-DPS (**c**), and D-DPS (**d**) in PS and PMMA films with increasing CA concentration from 0% to 25%. Dependence of *λ*_Total_ on Δ*f* for D–A (**e**) and D–A–D molecules (**f**) and linear fitting.

As shown in Figure 1a–d, the differed CT characteristics can be observed by Stokes shift, indicating the electrostatic stabilization through solvatochromic relationships such as the Lippert–Mataga equation [55]. In PS films without CA, P-TRZ, D-TRZ, P-DPS, and D-DPS exhibit Stokes shifts of 3738, 4395, 4524, and 4273 cm^−1^, respectively. Upon doping with 25 wt% CA, the corresponding values increase to 4711, 6000, 5363, and 5431 cm^−1^ (Table 2), resulting in Stokes shift changes (ΔS_0–25_^PS^) of 973, 1605, 839, and 1158 cm^−1^, respectively. For the D–A emitters, D-TRZ exhibits a larger Stokes shift than P-TRZ both in the absence and presence of CA, while the corresponding ΔS_0–25_^PS^ of D-TRZ is considerably higher than P-TRZ as well. This trend is consistent with the stronger dielectric response of D-TRZ and suggests stronger electrostatic interactions with the environment. By contrast, the D–A–D type D-DPS exhibits a comparable Stokes shift to that of P-DPS under the same dielectric constants (CA = 0 and CA = 25 wt%), with the donor electron-donating ability in the dielectric response being less pronounced than for the D–A counterparts.

More importantly, P-DPS (4524 cm^−1^ at CA = 0 and 5363 cm^−1^ at CA = 25 wt%) exhibits a larger Stokes shift than the corresponding D–A emitter P-TRZ (3738 cm^−1^ at CA = 0 and 4711 cm^−1^ at CA = 25 wt%), even though the TRZ-based D–A emitters possess stronger CT characteristics. This discrepancy may be attributed to the greater conformational flexibility of P-DPS, leading to additional SR and a red-shifted emission. A similar contrast is less pronounced for D-TRZ and D-DPS, which may arise from the strong dielectric response of D-TRZ and thus makes the respective contributions of electrostatic interactions in D-TRZ and SR in D-DPS more challenging to distinguish. Consequently, the Stokes shift in the involved DPS-based D–A–D emitters can hardly be barely explained by electrostatic stabilization alone. In addition, the involved emitters exhibit a further increased Stokes shift in PMMA films, consistent with the higher dielectric constants and the greater guest flexibility in PMMA (Table 2). Therefore, the Stokes shift cannot provide a complete assessment of multifaceted interactions involving both charge transfer (CT) and structural relaxation (SR) of guest emitters.

Furthermore, the Lippert–Mataga analysis (Figure S2 and Table S6) confirms that the effect of solvent polarity on the electrostatic stabilization of the four emitters is broadly consistent with that observed in solid films. Given the similar Onsager radius (*a*_w_) of the four emitters, a larger change in dipole moment (Δ*μ*) generally indicates a stronger electrostatic contribution to the multifaceted interactions. For the D–A emitters, D-TRZ exhibits a larger Δ*μ* (18.47 D) than P-TRZ (10.59 D), consistent with the corresponding ΔS_0–25_^PS^ in the solid films. For the D–A–D emitters, D-DPS shows only a slightly larger Δ*μ* (15.27 D) than P-DPS (13.67 D), also matching the solid-film data; thus, donor substitution has a weaker effect on the CT character in D–A–D than in the D–A emitters. Notably, P-DPS has a much larger Δ*μ* than P-TRZ (13.67 vs. 10.59 D), a difference more pronounced than in ΔS_0–25_^PS^ (973 vs. 839 cm^−1^), reflecting less constrained structural relaxation in solution. Nevertheless, D-TRZ still shows a larger Δ*μ* than D-DPS, indicating that the strong dielectric response of D-TRZ partially obscures a direct comparison between its electrostatic contribution and the structural relaxation contribution of D-DPS.

For evaluating the contribution of the excited-state SR, the internal reorganization energy of S_1_→S_0_ transition (*λ*_int_) was calculated for the involved emitters by summing up the contributions of all active vibrational modes in the MOMAP 2021A package [56,57,58,59]. As expected, the DPS-based D–A–D emitters exhibit markedly larger structural reorganization in S_1_→S_0_ transition (P-DPS: 2785 cm^−1^; D-DPS: 2338 cm^−1^) than the corresponding TRZ-based D–A counterparts (P-TRZ: 2074 cm^−1^; D-TRZ: 1687 cm^−1^), indicating extensive S_1_/S_0_ SR that can respond to the rigid environment.

Based on the analysis above, the D–A and D–A–D emitters can be distinguished by stronger CT and SR characteristics, respectively, providing a molecular basis for their different host–guest multifaceted interactions in doped films. For an integrated assessment of host–guest multifaceted interactions, a quantitative indicator is required to link the excited-state characteristics with the environmental response, for which the total reorganization energy (*λ*_Total_) was extracted from the experimentally measured steady-state absorption and emission spectra. In general, *λ*_Total_ includes the intramolecular contribution (*λ*_int_) arising from internal vibrational degrees of freedom and external contribution (*λ*_ext_) associated with the polarization response of the solute–environment interaction [60,61,62], which can be extracted as

(1) $$\lambda_{\mathrm{Total}}=\hbar \frac{\int_{0}^{\infty }\tilde{\omega }\left[\sigma_{a}\left(\tilde{\omega }\right)-\sigma_{f}\left(\tilde{\omega }\right)\right]d\tilde{\omega }}{\int_{0}^{\infty }\left[\sigma_{a}\left(\tilde{\omega }\right)+\sigma_{f}\left(\tilde{\omega }\right)\right]d\tilde{\omega }}$$

Here, *σ*_a_(*ῶ*) and *σ*_f_(*ῶ*) are the experimentally normalized absorption and emission spectra, respectively, and *ῶ* = *ω* − *ω*_eg_, where *ω*_eg_ is the frequency at the intersection of the absorption and emission spectra. The detailed data are listed in Tables S7 and S8.

To provide a quantitative depiction of the host–guest multifaceted interactions, the extracted values of *λ*_Total_ were plotted against the orientation polarizability Δ*f* (see Table S9) as shown in Figure 1e,f. Here, the dielectric constants were estimated from the CA doping concentration using published linear relationships between the CA content and the dielectric constant, i.e., *ε* = 2.45 + 0.120x for PS and *ε* = 3.41 + 0.219x for PMMA, where x is the CA concentration (wt%) [23]. In this picture, molecules that are highly sensitive to the dielectric environment exhibit a larger contribution from *λ*_ext_, thereby increasing *λ*_Total_, which is particularly pronounced for the D–A emitters with strong CT characteristics.

As shown in Figure 1e, D-TRZ (orange) exhibited a notably steeper *λ*_Total_~Δ*f* dependence than P-TRZ (yellow), indicating a stronger contribution of electrostatic interactions to *λ*_Total_ in D-TRZ, consistent with its larger Stokes shift. In other words, the host–guest electrostatic interactions can effectively enhance *λ*_ext_, causing its *λ*_Total_ to surpass that of P-TRZ, albeit D-TRZ possesses a smaller *λ*_int_ (1687 cm^−1^) than P-TRZ (2073 cm^−1^). Consequently, CT-dominant D–A emitters exhibited a cross pattern of *λ*_Total_~Δ*f* dependence due to their different responses to the increase of host dielectric constants.

In contrast, for SR-dominant D–A–D emitters that are less insensitive to the dielectric environment, the extracted *λ*_Total_ is primarily governed by the *λ*_int_ associated with S_1_/S_0_ SR, leading to a nearly parallel *λ*_Total_–Δ*f* pattern (Figure 1f). Despite D-DPS showing larger changes in Stokes shift than P-DPS (ΔS_0–25_^PS^: 1158 > 839 cm^−1^; ΔS_0–25_^PMMA^: 1050 > 705 cm^−1^), its *λ*_Total_ is considerably smaller, as demonstrated by the fitted line of P-DPS (green) being consistently above that of D-DPS (blue) in Figure 1f. Moreover, the minor contribution of *λ*_ext_ leads to a gentle increase in *λ*_Total_ with Δ*f*, without the crossing pattern.

**Figure 2:**
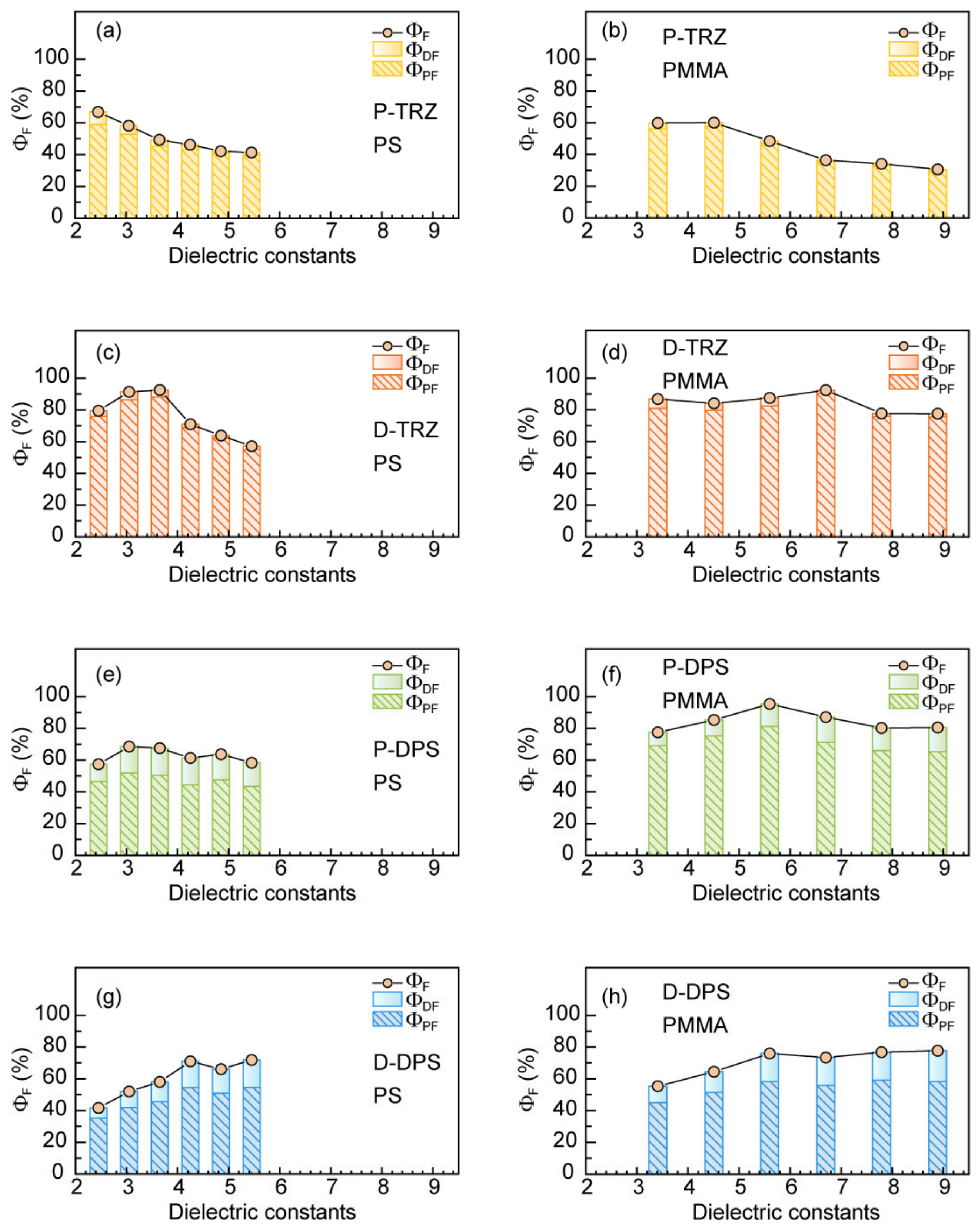
Measured fluorescence quantum yields (Φ_F_) of P-TRZ (**a**,**b**), D-TRZ (**c**,**d**), P-DPS (**e**,**f**), and D-DPS (**g**,**h**) in PS (left column) and PMMA (right column) films as a function of CA concentration. The contributions of prompt (Φ_PF_) and delayed (Φ_DF_) fluorescence are distinguished.

Although the analysis above indicates that D–A emitters are characterized by stronger CT characteristics, whereas D–A–D emitters exhibit more pronounced SR characteristics, the dependence of Φ_F_ with host dielectric constants exhibited more complex pattern. As shown in Figure 2a,b, P-TRZ shows a continuously decreasing Φ_F_ with increasing host dielectric constants, while D-DPS shows a totally reverse pattern (Figure 2g,h). Intriguingly, D-TRZ and P-DPS display similarly a non-monotonic pattern (Figure 2c–f), with Φ_F_ initially increasing and then decreasing as the CA concentration increases. As discussed above, *λ*_Total_ integrates the host–guest interactions, while Φ_F_ is a kinetic outcome determined by the competition among multiple excited state decay channels. Therefore, the diverse Φ_F_ trends imply that the discussion should be extended beyond a simple CT/SR ratio to the underlying rate constants, with Φ_F_ governed by prompt and delayed emission pathways. Accordingly, for the four emitters, Φ_PF_ and Φ_DF_ were separated from the measured Φ_F_ by exponential fitting of the prompt and delayed decay components [63], followed by weighting according to their integrated contributions, as exemplified by the PS-film data in Figures S3 and S4. This treatment provides the basis for the subsequent quantitative analysis of the relevant rate constants, with the corresponding rate equations summarized in Section S8 of the Supporting Information [9,64].

### 2.2. Delayed Fluorescence and Reverse Intersystem Crossing

The rate constants *k*_RISC_ greatly affect the efficiency of triplet-to-singlet conversion, therefore plays a key role in determining the Φ_DF_ of TADF emitters. To clarify the Φ_DF_ dependence on dielectric constants, *k*_RISC_ was analyzed within the semi-classical Marcus formalism, a well-established approximation of Fermi’s golden rule for ISC and RISC [65,66,67]:

(2) $$k_{\mathrm{RISC}}\;=\;\frac{{\left|\xi_{\mathrm{ST}}\right|}^{2}}{\hbar }\sqrt{\frac{2\pi }{\lambda k_{B}T_{\mathrm{eff}}}}\exp\left[-\frac{{\left(\Delta E_{\mathrm{ST}}-\lambda \right)}^{2}}{4\lambda k_{B}T_{\mathrm{eff}}}\right]$$

Here *ξ*_ST_ and Δ*E*_ST_ stand for the spin–orbit coupling matrix element and the singlet–triplet energy gap, respectively. Meanwhile, *λ* is the reorganization energy associated with RISC, while *T*_eff_ corresponds to the effective temperature. Due to the local excited (LE) nature, excitation energy of the T_1_ state is insensitive to host dielectric constants while the S_1_ (CT) state can be stabilized with the increasing of the CA concentration, for which the host dielectric constants are assumed to affect the energy gap (Δ*E*_ST_) rather than the relevant vibrational modes. Accordingly, Δ*E*_ST_ was adopted as the key parameter for describing the dielectric dependence of *k*_RISC_, by using estimated Δ*E*_ST_ rather than directly measured ones (Table S10). Specifically, Δ*E*_ST_ in PS films (Δ*E*_ST(CA0)_^PS^) was estimated by the adiabatic Δ*E*_ST_^*^ obtained from TD-DFT calculations, whereas Δ*E*_ST_ at other dielectric constants was approximated by assuming a nearly unchanged T_1_ level and a descent with the S_1_ energy shift.

**Figure 3:**
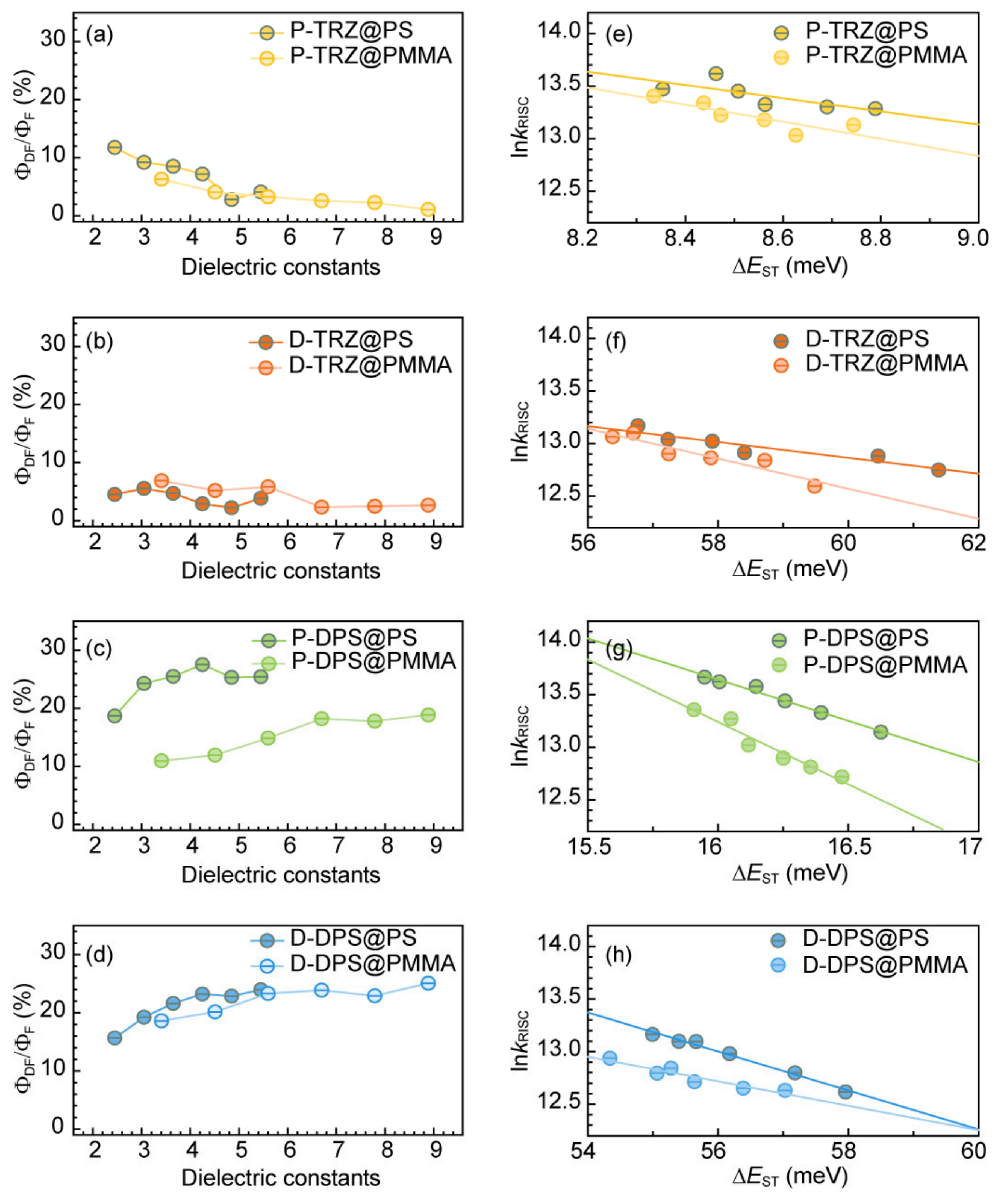
Contributions of the delayed fluorescence component (Φ_DF_/Φ_F_, left panels) and the corresponding ln*k*_RISC_ as a function of Δ*E*_ST_ (right panels) for P-TRZ (**a**,**e**), D-TRZ (**b**,**f**), P-DPS (**c**,**g**), and D-DPS (**d**,**h**) in PS and PMMA films with increasing CA concentration.

As shown in Figure 3a–d, the D–A–D emitters exhibit a gradual increase in the Φ_DF_/Φ_F_ ratio with increasing dielectric constants. This trend is attributed to the reduced Δ*E*_ST_ that can enhance both Φ_ISC_ and Φ_RISC_, thereby demonstrating that Φ_DF_ positively contributes to Φ_F_. In contrast, the Φ_DF_/Φ_F_ ratio of the D–A emitters remains nearly unchanged or even slight decreases with the Φ_DF_ contribution consistently below 10%, suggesting that the RISC channel is more effective in the D–A–D systems than in their D–A counterparts.

For further analysis, ln*k*_RISC_ was plotted against the estimated Δ*E*_ST(CA0–25)_ in PS and PMMA film as shown in Figure 3e–h. In all cases, ln*k*_RISC_ increased as Δ*E*_ST_ decreased, consistent with the expected dielectric stabilization of the CT (S_1_) state and the subsequent promotion of the RISC process (Table S10). This trend is further supported by the dielectric dependence of the delayed fluorescence lifetimes (*τ*_DF_), which decrease with increasing dielectric constants (Figure S5). Intriguingly, the involved emitters displayed similar *k*_RISC_ trend with Δ*E*_ST_ despite their markedly different Φ_DF_/Φ_F_ patterns with host dielectric constants. Therefore, the diverse Φ_F_ pattern observed among the four emitters (Figure 2) cannot be simply explained by variations in *k*_RISC_ due to the extremely low Φ_DF_/Φ_F_ ratio of the D–A emitters, i.e., the ISC/RISC cycling is insufficiently competitive against other singlet state deactivation pathways upon optical excitation (Table S10) [68,69]. Therefore, identifying the competitive decay channels of S_1_ state becomes essential for understanding the observed variation in Φ_F_ with host dielectric constants. Moreover, since the radiative decay (*k*_r_^S^) is mainly governed by S_1_⟶S_0_ transition dipole moment and is therefore much less sensitive to the dielectric environment, the key variable would be the singlet nonradiative decay rate (*k*_nr_^S^). This consideration directly motivates the following analysis.

### 2.3. Prompt Fluorescence and Nonradiative Decay Rate

As a time constant that has a significant impact on fluorescence efficiency, *k*_nr_^S^ (S_1_⟶S_0_) of organic emitters and the corresponding environmental effects have been widely discussed within the framework of Fermi’s golden rule [70,71,72,73,74]. Depending on S_1_/S_0_ electronic coupling, different formulism of *k*_nr_^S^ can be employed [75,76,77,78]. In this work, the involved TADF emitters exhibit large energy gaps (Δ*E*_S1→S0_) and considerable fluorescence efficiency, indicating they fall within the weak coupling limit, where *k*_nr_^S^ is given by

(3) $$k_{\mathrm{nr}}^{S}=\frac{C^{2}}{\hbar }\sqrt{\frac{2\pi }{\hbar \omega_{M}\Delta E_{S1\to S0}}}\exp\left\{-\frac{\Delta E_{S1\to S0}}{\hbar \omega_{M}}\left[\ln\left(\frac{\Delta E_{S1\to S0}}{l\lambda_{M}}\right)-1\right]\right\}$$

in which *λ*_M_ and *ω*_M_ represent the reorganization energy and vibrational frequency, respectively, for the specific vibrational modes coupled to the S_1_→S_0_ transition. However, since experimental results often imply a concerted vibronic coupling including multiple modes rather than an individual mode, *ω*_M_ can be replaced by the average effective frequency, *ω*_avg_ = ∑*_k_S_k_ω_k_*/∑*_k_S_k_* [72,79,80], in which *S_k_* is the Huang−Rhys factor of mode *k*. Accordingly, a smaller *ω*_avg_ indicates that the weighted distribution of vibronic coupling is more concentrated in the low-frequency region, and *S_k_* of modes in this region is generally higher than in the high-frequency modes [74,81]. As low-frequency modes are typically associated with collective motion, such as bond bending and dihedral twisting, enhanced contributions in the low-frequency region can lead to a larger internal reorganization energy and thus indicate a more pronounced S_1_/S_0_ SR characteristic [15,82,83,84,85].

**Figure 4:**
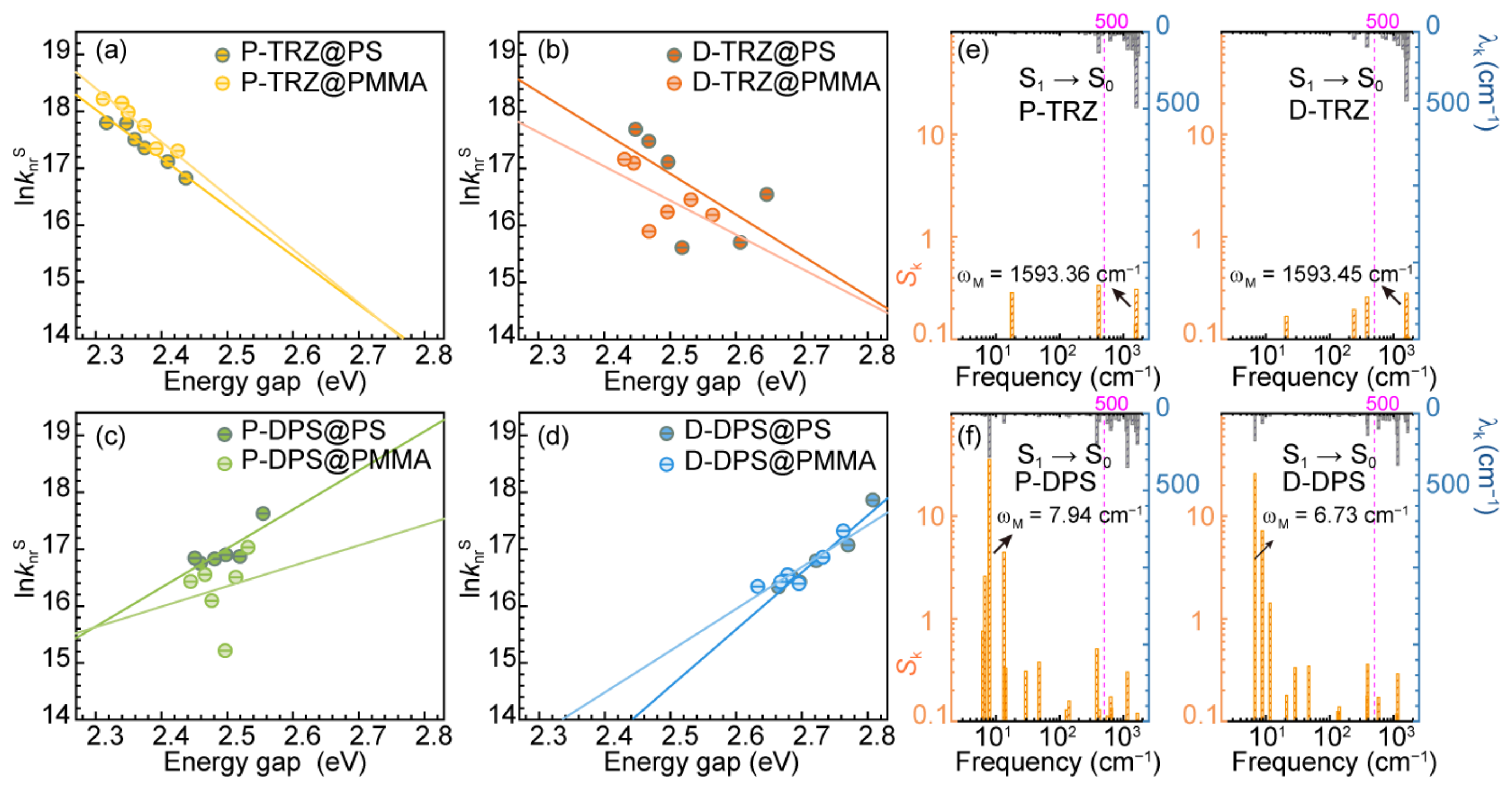
Dependence of *k*_nr_^S^ on S_1_⟶S_0_ energy gap for P-TRZ (**a**), D-TRZ (**b**), P-DPS (**c**), and D-DPS (**d**) in PS and PMMA films with increasing CA concentration. Huang–Rhys factors and reorganization energy contributions of each vibrational mode for D–A (**e**) and D–A–D emitters (**f**).

In a simplified energy gap law, *k*_nr_^S^ of organic emitters increases as Δ*E*_S1→S0_ decreases dominantly, i.e., *k*_nr_^S^∝e^−αΔ*E*^, in which the contribution of the vibrational effect is ignored and might lead to mistakes in systems with strong vibronic coupling for the S_1_⟶S_0_ transition. As shown in Figure 4a,b, ln*k*_nr_^S^ of the TRZ-based D–A emitters exhibited negative slope versus Δ*E*_S1→S0_, consistent with this simplified law. Intriguingly, however, for the DPS-based D–A–D emitters with enhanced conformational flexibility, a positive ln*k*_nr_^S^~Δ*E*_S1→S0_ slope was observed in both PS and PMMA matrices (Figure 4c,d), thereby deviating from the simplified energy gap law. The results are further supported by prompt fluorescence lifetime (*τ*_PF_) measurements, in which opposite trends of *τ*_PF_ with increasing dielectric constants were revealed for the D–A and D–A–D emitters (Figure S6). The observed dramatically different ln*k*_nr_^S^~Δ*E*_S1→S0_ dependence likely originates from the contributions of vibrational modes engaged in S_1_⟶S_0_ vibronic coupling (see Equation (3)), for which a detailed vibronic analysis on the S_1_⟶S_0_ transition of the involved emitters was performed and displayed in Figure 4e,f and Table 3.

For P-TRZ and D-TRZ, the reorganization energy contributions (∑*λ*_k_ < 10 cm^−1^) associated with low-frequency modes (*ω_k_* < 500 cm^−1^) are negligible compared to the total reorganization energy of S_1_⟶S_0_ transition (P-TRZ: 2073 cm^−1^; D-TRZ: 1687 cm^−1^, Figure 4e). The modes with the largest *λ*_k_ appear at *ω*_M_ ≈ 1593 cm^−1^, corresponding to stretching motion of the triazine acceptor (Table S11). Consequently, average effective frequency *ω*_avg_ values for P-TRZ and D-TRZ are 877 cm^−1^ and 896 cm^−1^, respectively, predominantly distributed in the high-frequency region. In contrast, the D–A–D emitters exhibit greatly enhanced vibronic coupling in the low-frequency region (Figure 4f). For the ultra-low frequency modes (*ω*_M_ = 7.94 cm^−1^ for P-DPS and 6.73 cm^−1^ for D-DPS), the *λ*_k_ values are two orders of magnitude higher (283.4 cm^−1^ and 115.2 cm^−1^, respectively) than those of the corresponding D–A emitters. These modes mainly involve out-of-plane wagging and the rotation of the donor groups, which significantly enhance the conformational flexibility of the D–A–D emitters (Table S11). Notably, low-frequency modes of D–A–D emitters exhibit more considerable Huang–Rhys factor (*S_k_* > 1) than those of the D–A emitters, indicating stronger coupling with S_1_⟶S_0_ transition. As a result, *ω*_avg_ with a major contribution from low-frequency modes is essential for describing the D–A–D emitters, with values as low as 58 cm^−1^ and 60 cm^−1^, indicating considerable conformational flexibility on S_1_ state.

**Figure 5:**
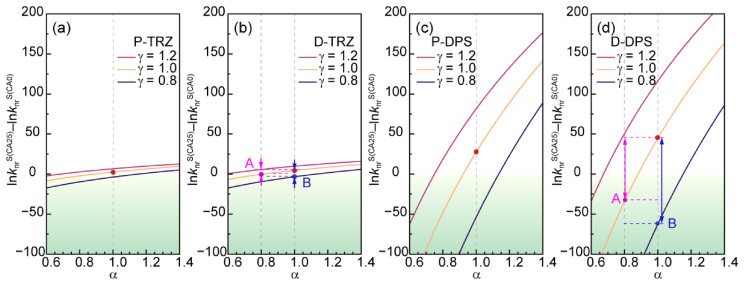
Effects of varying *λ* with coefficient α (*λ*^(CA25)^ = *αλ*^(CA0)^) and *ω*_avg_ with coefficient *γ* (*ω*_avg_^(CA25)^ = *γω*_avg_^(CA0)^) on ln*k*_nr_^S(CA25)^ − ln*k*_nr_^S(CA0)^ for P-TRZ (**a**), D-TRZ (**b**), P-DPS (**c**) and D-DPS (**d**). The green shaded region corresponds to deviation from the simplified energy gap law, i.e., *k*_nr_^S^∝e^−αΔ*E*^.

To analyze the concerted vibronic coupling on *k*_nr_^S^ within the framework of the weak coupling limit (Equation (3)), we calculated subtractive values (ln*k*_nr_^S(CA25)^ − ln*k*_nr_^S(CA0)^) to directly compare the response of *k*_nr_^S^ to the dielectric environment, with the constant *C*^2^ eliminated. In this treatment, positive values correspond to a responsive behavior consistent with the simplified energy gap law, i.e., *k*_nr_^S^∝e^−αΔ*E*^, whereas negative values indicate a deviation from it. Please note that such an analysis is highly challenging, the key point is that we cannot directly calculate the reorganization energy (*λ*) and average effective frequency (*ω*_avg_) in doped films at different CA%. Therefore, to account for changes in reorganization energy and average effective vibrational frequency in the doped films, we assumed *λ*^(CA0)^ and *ω*_avg_^(CA0)^ are equal to the calculated values, while two coefficients *α* and *γ* were introduced to multiply *λ* and *ω*_avg_ at CA% = 0, i.e., *λ*^(CA25)^ = *αλ*^(CA0)^ and *ω*_avg_^(CA25)^ = *γω*_avg_^(CA0)^. Here, an *α* or *γ* greater than 1 indicates an increase in *λ* or *ω*_avg_ in the dielectric environment, whereas *α* or *γ* less than 1 indicates a decrease. The analyzed results are shown in Figure 5, with the relevant data summarized in Table 3.

AS can be seen in Figure 5, the reorganization energy (*λ*) and average effective frequency (*ω*_avg_) involved in vibronic coupling can greatly affect ln*k*_nr_^S(CA25)^ − ln*k*_nr_^S(CA0)^, even dragging it into the green shaded region, i.e., the inverted region relative to the simplified energy gap law. In general, *α* < 1.0 and *γ* < 1.0 are more favorable for producing ln*k*_nr_^S(CA25)^ − ln*k*_nr_^S(CA0)^ < 0, corresponding to *λ*^(CA25)^ < *λ*^(CA0)^ and *ω*_avg_^(CA25)^ < *ω*_avg_^(CA0)^. This implies that the suppression of vibronic coupling in doped films, i.e., *λ* reducing and *ω*_avg_ shifting to a lower frequency, can offset the energy gap associated *k*_nr_^S^ increase and lead to reduced *k*_nr_^S^ in doped films with increased dielectric constants.

Intriguingly, the D–A and D–A–D emitters exhibit markedly different patterns of ln*k*_nr_^S(CA25)^ − ln*k*_nr_^S(CA0)^ with *α* and *γ*. For the D–A–D emitters, the steeper slopes lead to much larger variations in ln*k*_nr_^S(CA25)^ − ln*k*_nr_^S(CA0)^, indicating that *k*_nr_^S^ is highly sensitive to changes in the reorganization energy (described by the coefficient *α*). Furthermore, varying *ω*_avg_ (described by the coefficient *γ*) does not substantially alter the slope or range of ln*k*_nr_^S(CA25)^ − ln*k*_nr_^S(CA0)^, but shifts the pattern upward or downward relative to the yellow line (*γ* = 1.0). Overall, the striking contrast between the D–A and D–A–D type emitters lies in the slope, which can be traced to the key factor Δ*E*_S1→S0_/ℏ*ω*_avg_. Given that the Δ*E*_S1→S0_ values of the involved emitters are comparable, the observed differences in slope are primarily determined by *ω*_avg_. According to the vibrational analysis (Figure 4e,f), the *ω*_avg_ values of the D–A–D (~60 cm^−1^) and D–A (~900 cm^−1^) emitters differ by a factor of approximately 15, leading to a substantial difference in slope. Consequently, the larger Δ*E*_S1→S0_/ℏ*ω*_avg_ in the D–A–D emitters results in a steeper pattern of ln*k*_nr_^S(CA25)^ − ln*k*_nr_^S(CA0)^ with *α*, such that even minor changes in *λ*^(CA25)^ can lead to substantial variations in ln*k*_nr_^S(CA25)^ − ln*k*_nr_^S(CA0)^. This reveals that the D–A–D emitters dominated by low-frequency modes exhibit steeper pattern and are more prone to deviate from the simplified energy gap law, which is consistent with the experimental observations (Figure 4c,d). Furthermore, the individual contributions of each parameter can be summarized as follows:

(1) When both *λ* and *ω*_avg_ are assumed to be unchanged with the dielectric environment, the variation in the energy gap alone cannot reproduce the experimentally observed inverted behavior of the D–A–D emitters, as indicated by the red point in Figure 5. Under this condition, the theoretically predicted ln*k*_nr_^S(CA25)^ − ln*k*_nr_^S(CA0)^ values are 2.48, 4.54, 27.4, and 44.7 for P-TRZ, D-TRZ, P-DPS, and D-DPS, respectively, as listed in Table 3.

(2) When both Δ*E*_S1→S0_ and *λ* are allowed to vary, entry into the negative region becomes much easier for the D–A–D emitters because their steeper slopes require only a small decrease in *λ*. Point A represents the values of ln*k*_nr_^S(CA25)^ − ln*k*_nr_^S(CA0)^ upon a 20% decrease in the reorganization energy. Taking D-TRZ and D-DPS as examples, the value for D-TRZ decreases from 4.54 to −0.38, whereas that for D-DPS decreases from 44.7 to −34.1. Both cases therefore move from the positive region into the negative region, which is consistent with previous studies showing that reducing the reorganization energy through the suppression of the promoting modes can lead to a rapid decrease in *k*_nr_^S^ and increased fluorescence efficiency [40,72,83]. The effect is more pronounced for the D–A–D emitters, making them more likely to deviate from the energy gap law.

(3) When *ω*_avg_ is reduced by 20% (*γ* = 0.8, point B), all emitters can in principle exhibit inverted behavior, the corresponding values decrease to −3.39 and −62.5 for D-TRZ and D-DPS, respectively. These results indicate that a 20% reduction in *ω*_avg_ can more effectively suppress *k*_nr_^S^ than a comparable reduction in *λ*.

Overall, the concerted vibronic coupling effect on *k*_nr_^S^ is substantially stronger for the D–A–D emitters with high conformational flexibility, owing to their low-frequency dominated vibronic coupling and larger reorganization energies. Therefore, these results demonstrate that the dielectric dependence of *k*_nr_^S^ is not governed by the energy gap alone, but by the combined influence of the vibronic coupling of the S_1_⟶S_0_ transition, i.e., *λ* and *ω*_avg_. It should be noted that this tendency is identified for the present DPS-based D–A–D model emitters, and its extension to other emitters still requires a case-by-case assessment.

### 2.4. General Discussion on Multifaceted Interactions

Quantitative analysis indicates that the variation in Φ_F_ in doped films is primarily associated with changes in *k*_nr_^S^ upon optical excitation, where the delayed fluorescence contribution is much less considerable. Thus, the observed trends in Φ_F_ across the four emitters are intrinsically governed by the underlying rate constants, which are in turn influenced by the excited-state characteristics (CT vs. SR) of the emitters, and their multifaceted interactions with the dielectric environments, i.e., host–guest dipole–dipole interactions and conformational restraint.

For the D–A emitters that exhibit strong CT characteristics and relatively small *λ*_int_, vibronic coupling is dominated by high-frequency modes, resulting in large *ω*_avg_ values, small Δ*E*_S1→S0_/ℏ*ω*_avg_ ratios, and consequently gentle slopes in the ln*k*_nr_^S(CA25)^ − ln*k*_nr_^S(CA0)^ patterns. As a result, the *k*_nr_^S^ of these emitters readily adheres to the S_1_⟶S_0_ energy gap. Their multifaceted interactions are primarily influenced by *λ*_ext_, which is effectively enhanced by the host dielectric constants. Among them, P-TRZ closely follows this behavior, while D-TRZ exhibits a beneficial increase in Φ_F_ at low dielectric constants. This deviation likely stems from the stronger role of *λ*_ext_ in D-TRZ compared to P-TRZ in varying *λ*_Total_. At low CA doping concentrations, the increase in *λ*_ext_ is less pronounced, thereby suppressing the rise in *k*_nr_^S^ and creating a favorable outcome. Thus, for the D–A emitters, suppressing *k*_nr_^S^ requires minimizing *λ*_ext_ and avoiding a high dielectric environment.

By contrast, the D–A–D emitters possess pronounced S_1_/S_0_ SR and weak CT characteristics, endowing them with greater conformational flexibility. Their vibronic coupling is dominated by low-frequency modes, resulting in smaller *ω*_avg_ values, larger Δ*E*_S1→S0_/ℏ*ω*_avg_ ratios, and steeper slopes in ln*k*_nr_^S(CA25)^ − ln*k*_nr_^S(CA0)^ patterns. Consequently, their *k*_nr_^S^ values are equally affected by the energy gap and the vibronic effect. As reflected by the dominance of *λ*_int_, their multifaceted interactions display negligible dependence on *λ*_ext_. Among them, D-DPS closely follows this behavior, while P-DPS displays a detrimental decrease in Φ_F_ at high dielectric constants. This is likely because the higher *λ*_int_ of P-DPS amplifies the increase in reorganization energy under high dielectric constants, leading to an unacceptable rise of *k*_nr_^S^. Therefore, for the D–A–D emitters, minimizing *λ*_int_ and avoiding a high dielectric environment are essential as well to suppress *k*_nr_^S^. Overall, the combination of molecular rigidity and low dielectric environment is therefore established as a promising strategy for suppressing *k*_nr_^S^ and achieving high fluorescence efficiency of TADF emitters.

On this basis, the CT parameters, *λ*_int_, *λ*_Total_, and *ω*_avg_ may serve as physically meaningful descriptors for linking excited-state characteristics to the key rate constants and fluorescence efficiency. Although their applicability to other TADF emitters still requires a case-by-case assessment, the present work may offer a possible basis for future data-driven screening based on larger datasets.

## 3. Experimental and Calculational Methods

Materials and doped film preparation. P-TRZ [10-(4-(4,6-diphenyl-1,3,5-triazin-2-yl)phenyl)-10H-phenoxazine], P-DPS [10,10′-(4,4′-sulfonylbis(4,1-phenylene))bis(10H-phenoxazine)], D-TRZ [1-(4-(4,6-diphenyl-1,3,5-triazin-2-yl)phenyl)-9,9-dimethyl-9,10-dihydroacridine], and D-DPS [bis(4-(9,9-dimethyl-9,10-dihydroacridine)phenyl)sulfone] were obtained from Xi’an Polymer Light Technology Corp. (Xi’an, China). Polystyrene (PS, P107085, density = 1.047 g cm^−3^) and poly(methyl methacrylate) (PMMA, P141444, density = 1.18 g cm^−3^) were purchased from Sigma-Aldrich (Shanghai, China). Camphoric anhydride (CA) was purchased from Shanghai Standard Co., Ltd. (Shanghai, China). All chemicals were used without further purification. Deionized water with a resistivity of 18 MΩ·cm was used in all experiments. Polymer solutions (5 wt%) were obtained by dissolving 1 g of PS or PMMA in 12.84 mL of toluene. To prepare films with different CA contents, 8.2, 17.3, 27.5, 38.9, and 51.9 mg of CA were added to 2 mL portions of the polymer solution, corresponding to CA concentrations of 5, 10, 15, 20, and 25 wt%, respectively. The appropriate amount of each CA/polymer solution was then transferred to another vial containing the corresponding TADF emitter to afford a doping concentration of 1 wt%. The masses of the TADF emitters used were 1.574, 1.607, 1.644, 1.685, 1.731, and 1.783 mg, corresponding to CA concentrations from 0% to 25%, respectively. All solutions were prepared through a three-step dissolution process under stirring at appropriate temperatures. The resulting ternary solutions were spin-coated onto cleaned quartz substrates at 2000 rpm for 1 min, with an acceleration rate of 2000 rpm/s.

Spectroscopic Experiments. Steady-state UV/Vis absorption and fluorescence emission spectra were recorded on a HITACHI U-3900 spectrophotometer and a HITACHI F-4700 fluorescence spectrophotometer (Hitachi, Tokyo, Japan), respectively. The fluorescence emission spectra of the solid films and solutions were recorded under excitation wavelengths of 300 and 320 nm, respectively. Prompt and delayed fluorescence lifetimes were measured using a time-correlated single-photon counting (TCSPC) spectrometer (PTI QuantaMaster 800, HORIBA). The absolute photoluminescence quantum yields (Φ_F_) of the doped films were measured using an integrating sphere equipped with a standard excitation source at 330 nm.

Computational Methods. All electronic structure calculations of the TADF emitters were carried out using the Gaussian 16 software package [86]. The S_0_, S_1_ and T_1_ geometries of the TADF emitters were optimized at the PBE0/6-311g** level. All optimized structures were confirmed to be true minima by vibrational frequency analysis. Solvent effects were described using the polarizable continuum model (PCM) with toluene (*ε* = 2.37), employing equilibrium solvation for optimizations and nonequilibrium solvation for subsequent single-point calculations.

## 4. Conclusions

To summarize, this work establishes a quantitative framework for understanding host–guest multifaceted interactions in dielectric environments by employing rigid TRZ-based D–A and flexible DPS-based D–A–D TADF emitters in PS/PMMA:TADF:CA ternary films. For integrating CT and SR contributions, *λ*_Total_ emerges as an effective indicator enabling the analysis of excited state characteristics and rate constants to rationalize the diverse Φ_F_. The DPS-based D–A–D emitters with pronounced conformational flexibility exhibit considerable vibronic coupling dominated by low-frequency modes. Consequently, their *k*_nr_^S^ values are equally affected by the energy gap and the vibronic effect, while dipole–dipole interaction and conformational restraint should be considered in the host–guest interaction in doped films. By contrast, the TRZ-based D–A emitters possess greater molecular rigidity and weak vibronic coupling dominated by high-frequency modes, for which their interaction with dielectric environments is primarily dominated by the dipole–dipole interaction. Accordingly, the suppression of low-frequency vibronic contributions and the employment of a low dielectric environment represent a promising route toward highly efficient host–guest devices.

## Figures and Tables

**Table 1:** Control variables of molecules and environment in multifaceted interactions.

	Molecule	Environment
Charge transfer	PXZ or DMAC	CA doping concentration
Structural relaxation	D–A or D–A–D	PS or PMMA

**Table 2:** Stokes shift and corresponding changes in four emitters in PS and PMMA films at 0 and 25 wt% CA.

Stokes Shift (cm^−1^)	P-TRZ	D-TRZ	P-DPS	D-DPS
PS_(CA0)_	3738	4395	4524	4273
PS_(CA25)_	4711	6000	5363	5431
ΔS_0–25_^PS^	973	1605	839	1158
PMMA_(CA0)_	4747	5519	5727	5894
PMMA_(CA25)_	5662	6597	6432	6944
ΔS_0–25_^PMMA^	915	1079	705	1050

**Table 3:** Parameters for analyzing *k*_nr_^S^, including total Huang–Rhys factor (∑*_k_S_k_*), average effective frequency (*ω*_avg_), internal reorganization energy (*λ*_int_) and changes in ln*k*_nr_^S^ for PS and PMMA films.

	P-TRZ	D-TRZ	P-DPS	D-DPS
∑*_k_S_k_*	2.36	1.88	47.8	38.5
*ω*_avg_ (cm^−1^)	877	896	58	60
*λ*_int_ (cm^−1^)	2073	1687	2784	2338
PS: ln*k*_nr_^S(CA25)^ − ln*k*_nr_^S(CA0)^	0.97	1.14	−0.78	−1.53
Cal.: ln*k*_nr_^S(CA25)^ − ln*k*_nr_^S(CA0)^	2.48	4.54	27.4	44.7
PMMA: ln*k*_nr_^S(CA25)^ − ln*k*_nr_^S(CA0)^	0.92	0.97	−0.60	−0.98

## Data Availability

Data is contained within the article or Supplementary Materials.

## Supplementary Materials

The following supporting information can be downloaded at: https://www.mdpi.com/article/10.3390/molecules31101581/s1, Figure S1: Steady-state UV–vis absorption (lines) and fluorescence (area) spectra of P-TRZ (a), D-TRZ (b), P-DPS (c), and D-DPS (d) in toluene solvent (10^−5^ mol·L^−1^); Table S1. The calculated vertical (*E*) emission energy (in eV) comparison with corresponding experimental data of the four TADF emitters; Table S2: Experimental fluorescence energies measured in PS films and toluene solutions, together with the calculated vertical (*E*) and adiabatic energy (*E**) of the singlet state (S_1_) of the four TADF emitters; Table S3: Calculated CT parameters from hole–electron analysis of the four TADF emitters by TD-DFT (PBE0/6-311g**); Table S4: The DFT (PBE0/6-311g**) calculated vertical excited-states (S_1_) for investigated four emitters; Table S5: The DFT (PBE0/6-311g**) calculated visualized distribution of frontier molecular orbitals (HOMO to LUMO) for investigated four emitters; Figure S2: Lippert–Mataga plots of Stokes shifts for P-TRZ (a), D-TRZ (b), P-DPS (c) and D-DPS (d); Table S6: The calculated data for Lippert–Mataga relationship; Table S7: Calculated total reorganization energy (*λ*_Total_) for four TADF emitters in the PS films; Table S8: Calculated total reorganization energy (*λ*_Total_) for four TADF emitters in the PMMA films; Table S9: The dielectric constants of the PS and PMMA matrices with different CA concentrations were obtained via linear fitting; Figure S3: TCSPC decays of delayed fluorescence for P-TRZ (a–f) and D-TRZ (g–l) in PS films with different CA concentrations. Black squares are the experimental data, red lines are the fits, and blue and green lines are the individual fitted components. Insets summarize the fitting parameters and R^2^ values; Figure S4: TCSPC decays of delayed fluorescence for P-DPS (a–f) and D-DPS (g–l) in PS films with different CA concentrations. Black squares are the experimental data, red lines are the fits, and blue and green lines are the individual fitted components. Insets summarize the fitting parameters and R^2^ values; Figure S5: TCSPC measurements of delayed fluorescence and the corresponding *τ*_DF_ trends for P-TRZ (a,c), D-TRZ (b,d), P-DPS (e,g), and D-DPS (f,h) in PS and PMMA films with increasing CA concentration; Table S10: TD-DFT calculated adiabatic singlet–triplet energy gaps (Δ*E*_ST_^*^) and spin–orbit coupling elements (*ξ*_ST_), and the corresponding Δ*E*_ST_ values in PS and PMMA film; Figure S6: TCSPC measurement of prompt fluorescence and a tendency of *τ*_PF_ for P-TRZ (a,c), D-TRZ (b,d), P-DPS (e,g), and D-DPS (f,h) in PS and PMMA films with increasing CA concentration; Table S11: The dominant modes displayed in Huang–Rhys.

## Abbreviations

The following abbreviations are used in this manuscript:

CT	charge transfer
SR	structural relaxation
CA	camphoric anhydride
PS	polystyrene
PMMA	poly(methyl methacrylate)
P-TRZ	10-(4-(4,6-diphenyl-1,3,5-triazin-2-yl)phenyl)-10H-phenoxazine
D-TRZ	10-(4-(4,6-Diphenyl-1,3,5-triazin-2-yl)phenyl)-9,9-dimethyl-9,10-dihydroacridine
P-DPS	10-(4-(4-(10H-Phenoxazin-10-yl)phenylsulfonyl)phenyl)-10H-phenoxazine
D-DPS	10,10′-(4,4′-Sulfonylbis(4,1-phenylene))bis(9,9-dimethyl-9,10-dihydroacridine
LE	local excited
*E*(S_1_)	energy of the first singlet excited state
Δ*μ*	excited-state dipole-moment changes
*k*_nr_^S^	nonradiative decay rate
*k*_RISC_	reverse intersystem crossing
Φ_F_	fluorescence quantum yield
Φ_DF_	delayed fluorescence
Φ_PF_	prompt fluorescence
*τ*_DF_	delayed fluorescence lifetimes
*τ*_P__F_	prompt fluorescence lifetimes
*λ*_Total_	total reorganization energy
*λ*_int_	internal reorganization energy
*λ*_ext_	external reorganization energy
*λ*_M_	specific reorganization energy
ΔS_0–25_^PS^	change in Stokes shift of PS films from 0 wt% to 25 wt% CA
ΔS_0–25_^PMMA^	change in Stokes shift in PMMA films from 0 wt% to 25 wt% CA
*ξ*_ST_	spin–orbit coupling matrix
Δ*E*_ST_	singlet–triplet energy gap
Δ*E*_ST_^*^	adiabatic energy gap from TD-DFT
Δ*E*_ST(CA0)_^PS^	approximate adiabatic energy gap
Δ*E*_S1→S0_	energy gap between S_1_ and S_0_
*ω*_avg_	average effective frequency
*ω_k_*	vibrational frequency of mode *k*
*S_k_*	the Huang−Rhys factor of mode *k*
ln*k*_nr_^S(CA25)^ − ln*k*_nr_^S(CA0)^	change in the singlet nonradiative decay rate from 0 wt% to 25 wt% CA
*α*	changes in reorganization energy in doped films
*γ*	changes in average effective vibrational frequency in doped films
