# Supplementary material for: Multifaceted Interactions of Thermally Activated Delayed Fluorescent Emitters with Dielectric Environments: Charge Transfer vs. Structural Relaxation
Source: Molecules. 2026 May 9;31(10):1581. doi: 10.3390/molecules31101581 (PMC13209165; doi:10.3390/molecules31101581)
Supplement: Supplementary file 1 [file molecules-31-01581-s001.zip › molecules-4258791-supplementary.pdf]

Supporting Information

Multifaceted Interaction of Thermally Activated Delayed Fluorescent Emitters with Dielectric Environments: Charge Transfer *vs.* Structural Relaxation

Yiran Tian [1], Yaxin Wang [1,†], Yixuan Gao [1,⸸], Zilong Guo [2,‡], Shaowen Chu [1], Yonghang Li [1], Yandong Han [2], Wensheng Yang [2,1,] and Xiaonan Ma [1,*]*

1 Institute of Molecular Plus, Tianjin University, Tianjin 300072, China;
2 Engineering Research Center for Nanomaterials, Henan University, Kaifeng 475004, China;
† Current address: School of Science and Engineering, The Chinese University of Hong Kong, Shenzhen 518172, China;
⸸ Current address: North China Branch of SINOPEC Marketing Co., Ltd., Tianjin 300072, China;
‡ Current address: School of Materials Science and Engineering, Changchun University of Science and Technology, Changchun 130022, China.
* Correspondence: wsyang@henu.edu.cn (W.Y.); xiaonanma@tju.edu.cn (X.M.)

Content

Section		Page
S1	Spectrum experiment and TD-DFT calculation.	3
	Figure S1	3
	Table S1	4
	Table S2	5
S2	Calculated charge transfer parameters from hole–electron analysis.	6
	Table S3	6
S3	Vertical excited states and frontier orbitals.	7
	Table S4	7
	Table S5	8
S4	Relative parameters of Lippert–Mataga relationship.	9
	Figure S2	9
	Table S6	10
S5	λ_{Total} calculated from spectra.	11
	Table S7	11
	Table S8	12
S6	Dielectric constants.	13
	Table S9	13
S7	Extraction of Φ_{PF} and Φ_{DF} by exponential decay function.	14
	Figure S3	15
	Figure S4	16
S8	Key rate constants.	17
S9	Delayed lifetime on TCSPC method and fitting value.	18
	Figure S5	18
S10	Estimated ΔE_{ST} and Relevant parameters of the Marcus theory.	19
	Table S10	19
S11	Prompt lifetime on TCSPC method and fitting value.	20
	Figure S6	20
S12	Vibrational modes.	21
	Table S11	21

S1. Spectrum experiment and TD-DFT calculation.

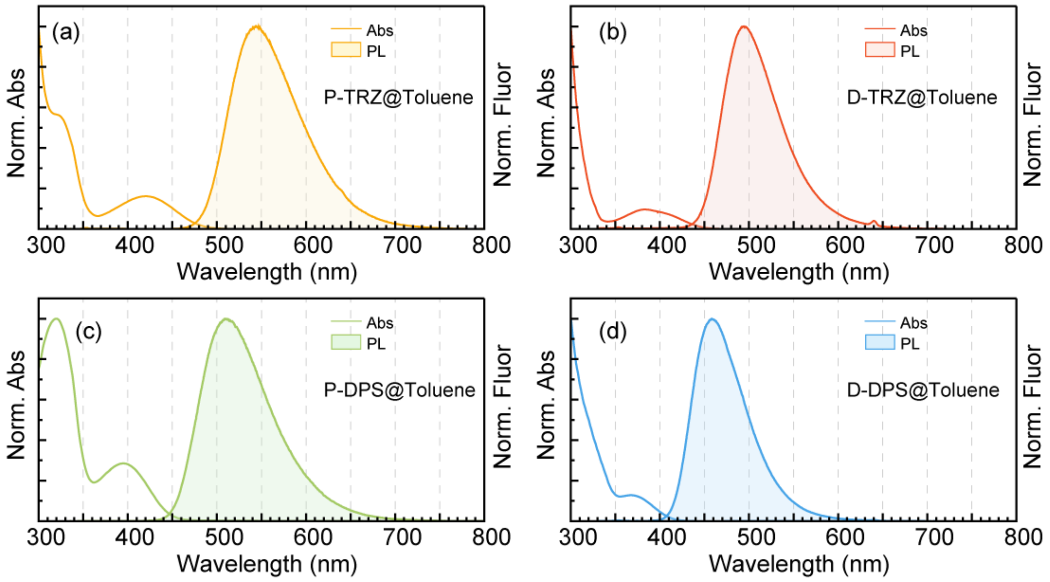

Figure S1. Steady-state UV–vis absorption (lines) and fluorescence (area) spectra recorded under 320 nm of P-TRZ (a), D-TRZ (b), P-DPS (c), and D-DPS (d) in toluene solvent (10^{-5} mol·L^{-1}).

Table S1. The calculated vertical (E) emission energy (in eV) comparison with corresponding experimental data of the four TADF emitters.

	P-TRZ		D-TRZ	
	value	error	value	error
E_{PL}^{TOL}/eV	2.28	–	2.51	–
TPSSh	1.82	−0.46	2.09	−0.42
B3LYP	2.19	−0.09	2.45	−0.06
PBE0	2.38	0.10	2.65	0.14
BMK	2.96	0.68	3.20	0.69
M06-2X	2.68	0.40	2.98	0.47
CAM-B3LYP	3.44	1.16	3.63	1.12
	P-DPS		D-DPS	
	value	error	value	error
E_{PL}^{TOL}/eV	2.45	–	2.71	–
TPSSh	2.15	−0.30	2.44	−0.27
B3LYP	2.47	0.02	2.76	0.05
PBE0	2.63	0.18	2.92	0.21
BMK	3.21	0.76	3.44	0.73
M06-2X	3.56	1.11	3.63	0.92
CAM-B3LYP	3.55	1.10	3.76	1.05

Table S2. Experimental fluorescence energies measured in PS films and toluene solutions, together with the calculated vertical (*E*) and adiabatic energy (*E**) of the singlet state (S_1) of the four TADF emitters.

		Experiment		DFT/TD-DFT	
Types	Materials	E_{PL}^{PS}/eV	E_{PL}^{TOL}/eV	$E(S_1)$/eV	$E^*(S_1)$/eV
D-A	P-TRZ	2.44	2.28	2.38	2.13
	D-TRZ	2.65	2.51	2.64	2.43
D-A-D	P-DPS	2.55	2.45	2.63	2.33
	D-DPS	2.81	2.71	2.92	2.67

S2. Calculated charge transfer parameters from hole–electron analysis.

Table S3. Calculated CT parameters from hole–electron analysis of the four TADF emitters by TD-DFT (PBE0/6-311g**).

Types	Materials	Excited-states	CT%	S_r	D (Å)	H (Å)	H_{CT}	t (Å)
D-A	P-TRZ	S_1	91.44	0.121	6.174	2.828	1.954	4.220
	D-TRZ	S_1	90.75	0.133	6.028	2.887	1.982	4.047
D-A-D	P-DPS	S_1	87.69	0.184	2.530	4.647	1.531	0.998
	D-DPS	S_1	87.34	0.178	2.462	4.594	1.564	0.898

The whole indexes of hole-electron analysis were descried in Multiwfn guideline[52,53]. To characterize the overlap between the hole and electron distributions, the S_r index is defined as follows:

$$S_r \text{ index} = \int S_r(\mathbf{r})d\mathbf{r} \equiv \int \sqrt{\rho^{hole}(\mathbf{r})\rho^{ele}(\mathbf{r})}d\mathbf{r} \quad (1)$$

The centroid can be calculated to reveal the most representative positions of the hole and electron distributions. The total magnitude of the CT length is referred to as the D index:

$$D \text{ index} = |D| \equiv \sqrt{(D_x)^2 + (D_y)^2 + (D_z)^2} \quad (2)$$

The H index measures the average spatial extent of the hole and electron distributions in all direction, H_{CT} represents the extent along the CT direction.

$$H \text{ index} = \frac{|\sigma_{hole}| + |\sigma_{ele}|}{2} \quad (3)$$

$$H_{CT} = |\mathbf{H} \cdot \boldsymbol{\mu}_{CT}| \quad (4)$$

The t index is designed to measure separation degree of hole and electron in CT direction:

$$t \text{ index} = D \text{ index} - H_{CT} \quad (5)$$

S3. Vertical excited states and frontier orbitals.

Table S4. The DFT (PBE0/6-311g**) calculated vertical excited-states (S_1) for investigated four emitters.

	P-TRZ			D-TRZ		
	Excitation energy (eV)	Transitions	Oscillator strength	Excitation energy (eV)	Transitions	Oscillator strength
S_1	2.379	H → L (97%)	< 0.0001	2.635	H → L (96.9%)	0.0001
	P-DPS			D-DPS		
S_1	2.629	H−1 → L (92.6%) H → L+2 (6.2%)	0.0044	2.918	H → L (92.4%) H−1 → L+2 (6.7%)	< 0.0001

Table S5. The DFT (PBE0/6-311g**) calculated visualized distribution of frontier molecular orbitals (HOMO to LUMO) for investigated four emitters.

	P-TRZ	D-TRZ	P-DPS	D-DPS
LUMO+2	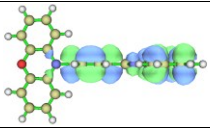	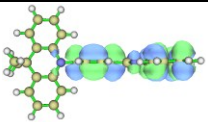	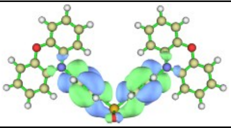	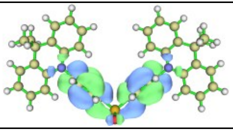
LUMO	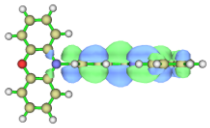	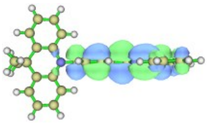	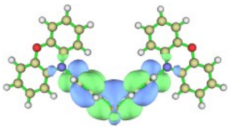	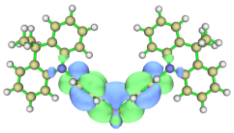
HOMO	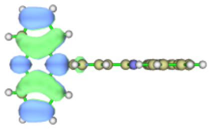	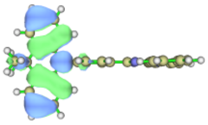	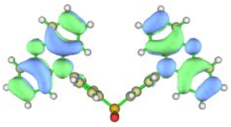	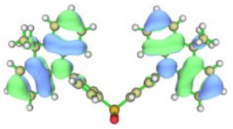
HOMO−1	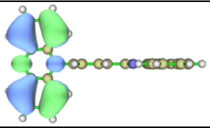	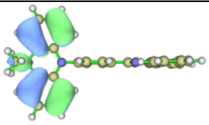	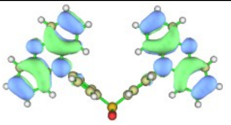	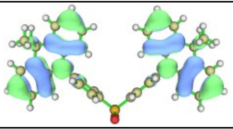

S4. Relative parameters of Lippert–Mataga relationship.

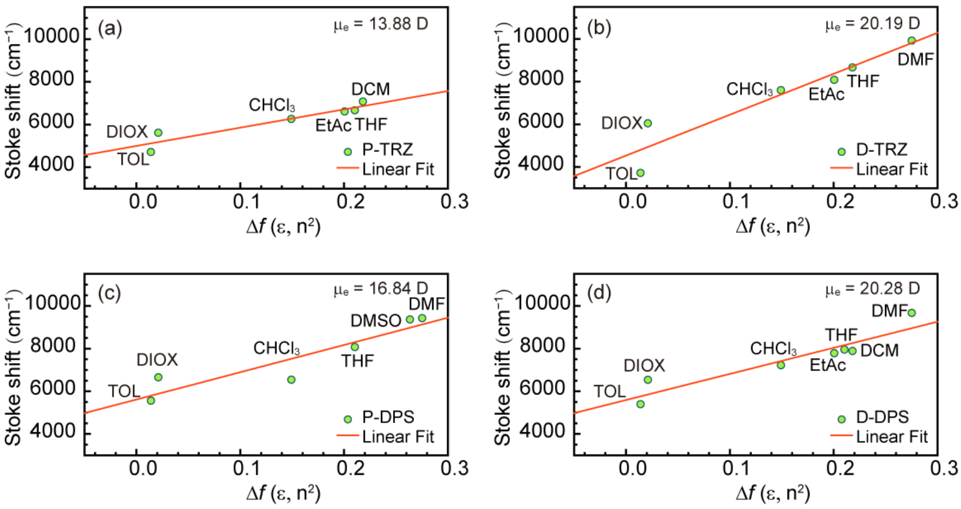

Figure S2. Lippert–Mataga plots of Stokes shifts for P-TRZ (a), D-TRZ (b), P-DPS (c) and D-DPS (d).

Table S6. The calculated data for Lippert–Mataga relationship.

	P-TRZ	D-TRZ	P-DPS	D-DPS
a_w (Å)	5.09	5.64	6.27	6.63
μ_g (D)	3.29	1.72	3.17	5.01
$\Delta\mu$ (D)	10.59	18.47	13.67	15.27
μ_e (D)	13.88	20.19	16.84	20.28

S5. λ_{Total} calculated from spectra.

Fleming et al. presented an accurate and easily applicable method to estimate λ_{Total} from the absorption and fluorescence spectra, as described above. Here, $\sigma_a(\tilde{\omega})$ and $\sigma_f(\tilde{\omega})$ represent the normalized absorption and emission spectra, respectively. The quantity $\tilde{\omega}$ is defined as $\tilde{\omega} = \omega - \omega_{eg}$, where ω_{eg} corresponds to the frequency at which the absorption and emission spectra intersect, with values listed in Tables S7–S8. The sole assumption underlying this approach is that the polarization fluctuations of the dielectric medium follow Gaussian statistics, which is valid for the present study.

Table S7. Calculated total reorganization energy (λ_{Total}) for four TADF emitters in the PS films.

	ω_{eg} (10^{14} s^{-1})				λ_{Total} (cm^{-1})			
	P-TRZ	P-DPS	D-TRZ	D-DPS	P-TRZ	P-DPS	D-TRZ	D-DPS
PS / 0 wt% CA	6.33	6.72	6.83	7.32	3771	3766	3553	3056
PS / 5 wt% CA	6.32	6.67	6.81	7.26	3796	3895	3622	3208
PS / 10 wt% CA	6.29	6.63	6.75	7.22	3864	4032	3803	3341
PS / 15 wt% CA	6.27	6.60	6.71	7.19	3936	4074	3904	3438
PS / 20 wt% CA	6.26	6.57	6.67	7.18	3981	4172	4022	3446
PS / 25 wt% CA	6.24	6.56	6.65	7.17	4042	4177	4060	3481

Table S8. Calculated total reorganization energy (λ_{Total}) for four TADF emitters in PMMA films.

	ω_{eg} (10^{14} s^{-1})				λ_{Total} (cm^{-1})			
	P-TRZ	P-DPS	D-TRZ	D-DPS	P-TRZ	P-DPS	D-TRZ	D-DPS
PMMA / 0 wt% CA	6.43	6.78	6.87	7.36	3845	4331	4051	3611
PMMA / 5 wt% CA	6.39	6.76	6.84	7.33	3981	4367	4154	3720
PMMA / 10 wt% CA	6.37	6.72	6.75	7.29	4020	4538	4400	3853
PMMA / 15 wt% CA	6.35	6.69	6.72	7.26	4072	4587	4494	3923
PMMA / 20 wt% CA	6.32	6.65	6.68	7.25	4131	4699	4615	3951
PMMA / 25 wt% CA	6.30	6.64	6.64	7.21	4229	4702	4714	4089

S6. Dielectric constants.

Serevičius et al. proposed a powerful CELIV technology to access the relationship between the dielectric constant and CA-doped concentration[23]. In this work, the published linear functions derived from least-squares fitting were adopted to estimate the dielectric constants. The calculated values are summarized below:

Table S9. The dielectric constants of the PS and PMMA matrices with different CA concentrations were obtained via linear fitting.

x (CA wt%)	PS: $\varepsilon = 2.45 + 0.120x$	PMMA: $\varepsilon = 3.41 + 0.219x$
	ε	ε
0	2.45	3.41
5	3.05	4.505
10	3.65	5.6
15	4.25	6.695
20	4.85	7.79
25	5.45	8.885

S7. Extraction of Φ_{PF} and Φ_{DF} by exponential decay function.

To separate the prompt and delayed contributions to the absolute photoluminescence quantum yield (Φ_F), the time-resolved decay is usually well fitted by the sum of two or three exponentials, describing the Φ_{PF} and Φ_{DF} decay[63]:

$$I_{\mathrm{PF}}(t) = \sum_i A_i \exp\left(-\frac{t}{\tau_i}\right) \tag{6}$$

$$I_{\mathrm{DF}}(t) = \sum_j A_j \exp\left(-\frac{t}{\tau_j}\right) \tag{7}$$

Where A_i, τ_i and A_j, τ_j are fitting coefficients describing the prompt and delayed decay component, respectively. Where I_{PF} and I_{DF} denote the integrated intensities of the prompt and delayed emission components, respectively. The total photoluminescence quantum yield (Φ_F) is

$$\Phi_{\mathrm{F}} = \Phi_{\mathrm{PF}} + \Phi_{\mathrm{DF}} \tag{8}$$

The ratio of the delayed to prompt fluorescence contributions is given by:

$$\Phi_{\mathrm{DF}}/\Phi_{\mathrm{PF}} = \frac{I_{\mathrm{DF}}}{I_{\mathrm{PF}}} \tag{9}$$

Using the experimentally measured absolute Φ_F together with the ratio from Eq. (9), the values of Φ_{PF} and Φ_{DF} were obtained by weighting the total emission according to the integrated prompt and delayed contributions. The corresponding rate constants were then derived from these experimentally determined quantities.

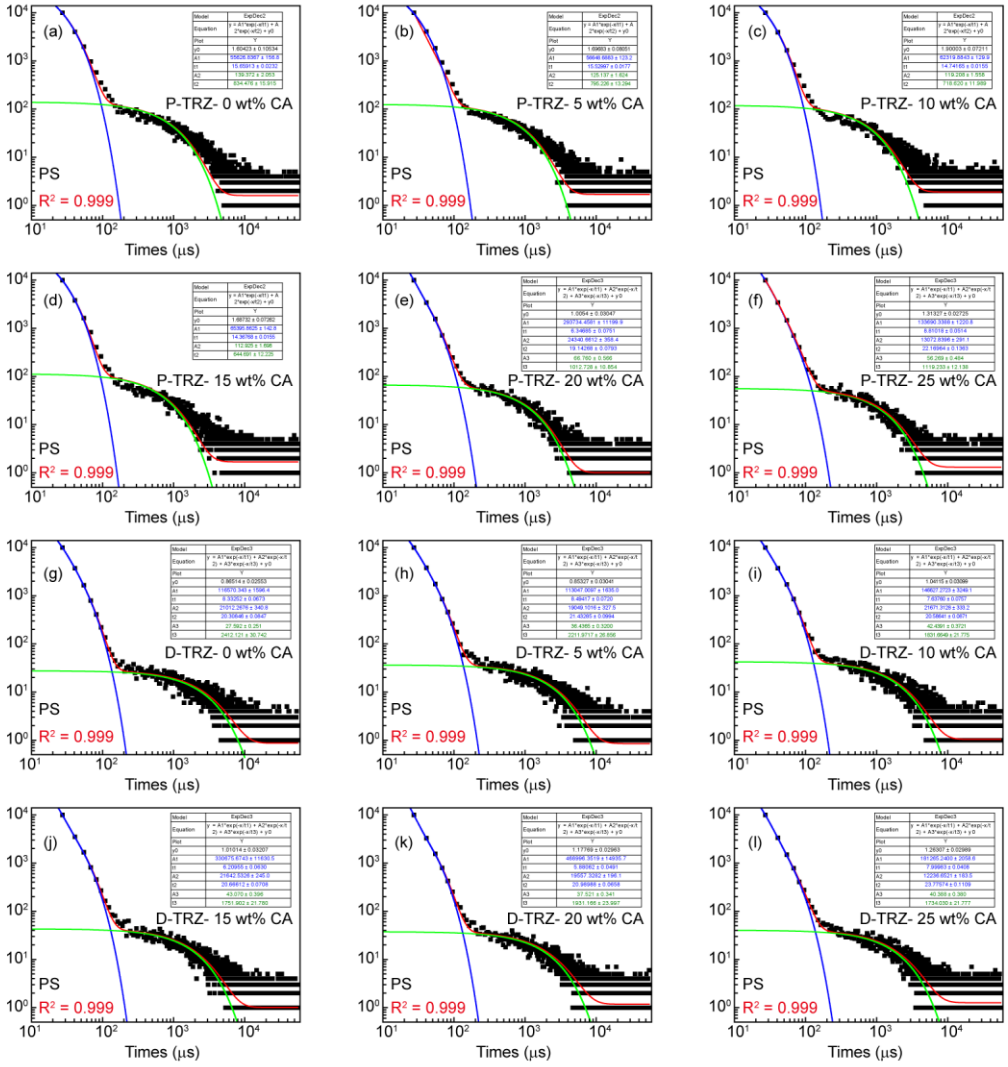

Figure S3. TCSPC decays of delayed fluorescence for P-TRZ (a-f) and D-TRZ (g-l) in PS films with different CA concentrations. Black squares are the experimental data, red lines are the fits, and blue and green lines are the individual fitted components. Insets summarize the fitting parameters and R^2 values.

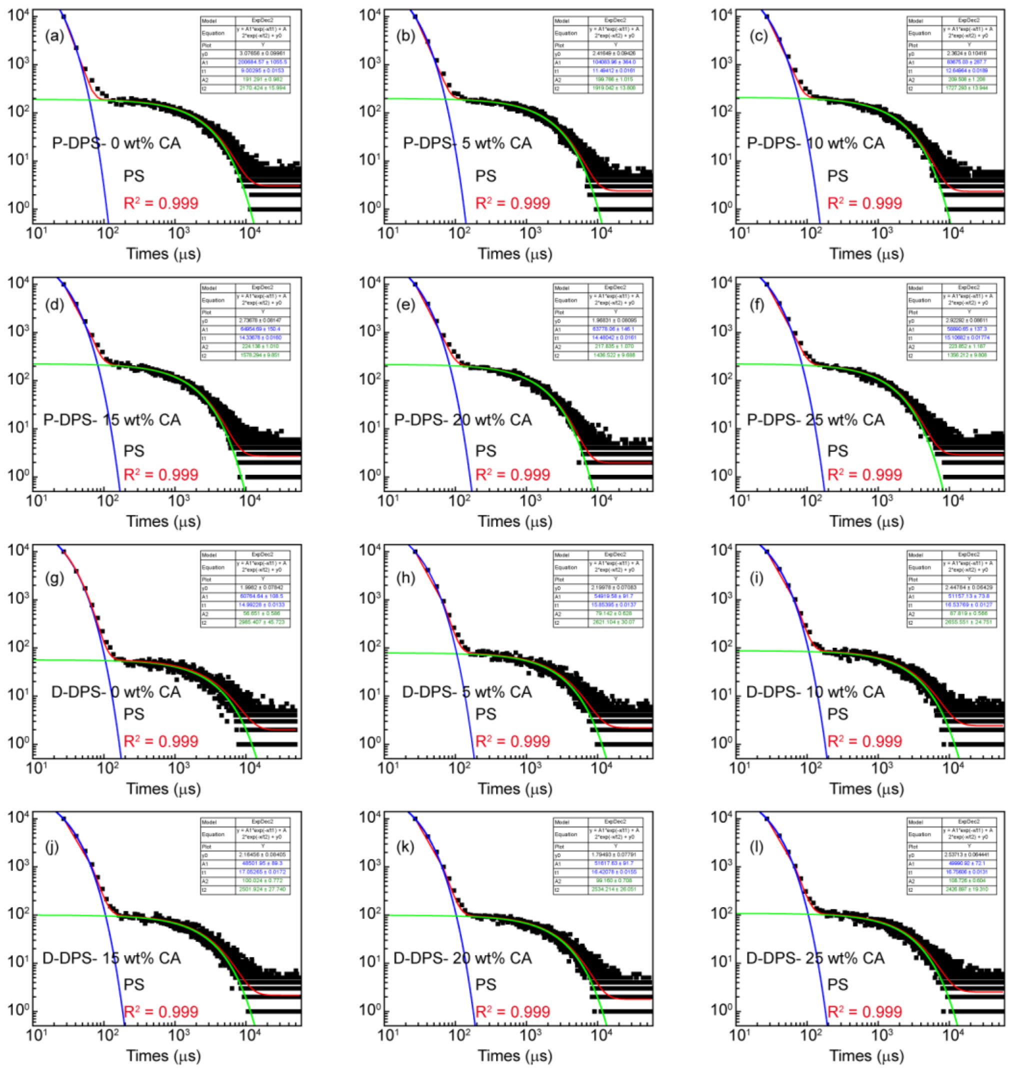

Figure S4. TCSPC decays of delayed fluorescence for P-DPS (a-f) and D-DPS (g-l) in PS films with different CA concentrations. Black squares are the experimental data, red lines are the fits, and blue and green lines are the individual fitted components. Insets summarize the fitting parameters and R^2 values.

S8. Key rate constants.

The rate constants of radiative decay (k_r) and nonradiative singlet decay (k_{nr}^S) from the S_1 to S_0 states, together with the rate constants of intersystem crossing (k_{ISC}) and reverse intersystem crossing (k_{RISC}), were calculated using the following six equations[9,64]:

$$k_{PF} = k_r + k_{nr}^S + k_{ISC} = \frac{1}{\tau_{PF}} \tag{10}$$

$$k_{DF} = (1 - \Phi_{ISC})k_{RISC} = \frac{1}{\tau_{DF}} \tag{11}$$

$$\Phi_{ISC} = \frac{k_{ISC}}{k_r + k_{nr}^S + k_{ISC}} \tag{12}$$

$$\Phi_{RISC} = \frac{k_{RISC}}{k_{nr}^T + k_{RISC}} \tag{13}$$

$$k_{ISC} = \frac{k_{PF}k_{DF}\Phi_{DF}}{k_{RISC}\Phi_{PF}} \tag{14}$$

$$k_{RISC} = \frac{k_{PF}k_{DF}\Phi_F}{k_r} \tag{15}$$

Here, τ_{PF} and τ_{DF} represent the prompt and delayed fluorescence lifetimes, respectively, as obtained from TCSPC measurements. Similarly, k_{PF} and k_{DF} denote the decay rate constants of prompt and delayed fluorescence, respectively. Φ_{PF} and Φ_{DF} represent the prompt and delayed fluorescence photoluminescence quantum yields, respectively, which were extracted by weighting the total Φ_F with the separately integrated prompt and delayed exponential decay components. In the present analysis, nonradiative triplet decay rate (k_{nr}^T) was neglected because the delayed fluorescence contribution to Φ_F is relatively small.

S9. Delayed lifetime on TCSPC method and fitting value.

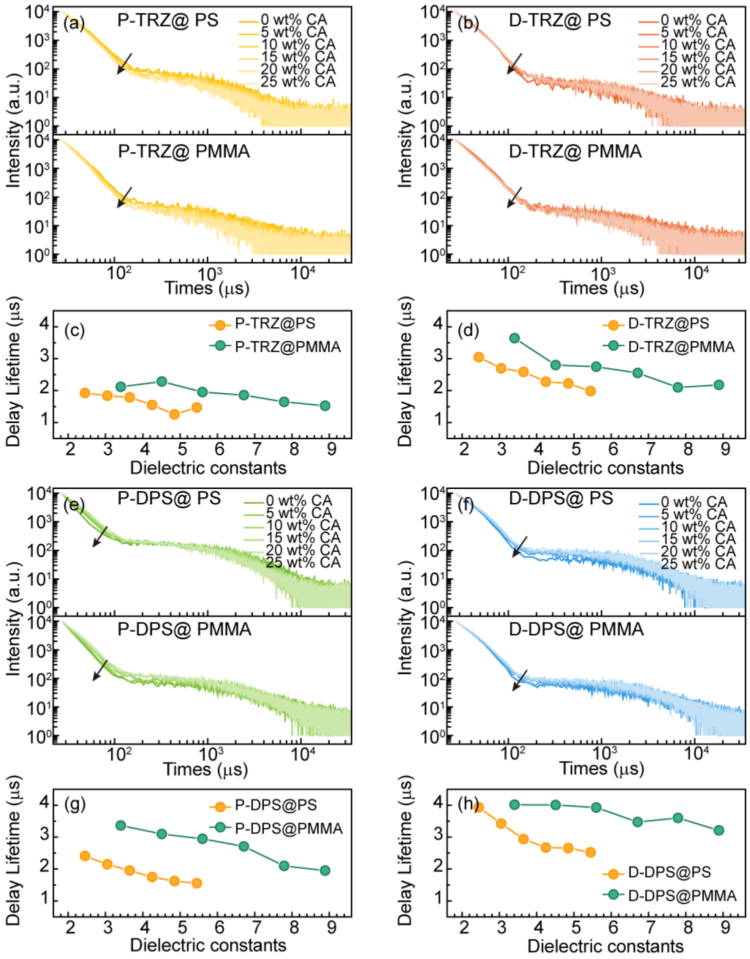

Figure S5. TCSPC measurements of delayed fluorescence and the corresponding τ_{DF} trends for P-TRZ (a, c), D-TRZ (b, d), P-DPS (e, g), and D-DPS (f, h) in PS and PMMA films with increasing CA concentration.

S10. Estimated ΔE_{ST} and Relevant parameters of the Marcus theory.

Table S10. TD-DFT calculated adiabatic singlet–triplet energy gaps (ΔE_{ST}^*) and spin–orbit coupling elements (ξ_{ST}), and the corresponding $\Delta E_{ST(CA0-25)}$ values in PS and PMMA film.

	P-TRZ	D-TRZ	P-DPS	D-DPS
ΔE_{ST}^* (meV)	8.80	61.4	16.6	58.0
ξ_{ST} (cm^{-1})	0.001	0.002	0.020	0.038
$\Delta E_{ST(CA0)}^{PS}$ (meV)	8.80	61.4	16.6	58.0
$\Delta E_{ST(CA5)}^{PS}$ (meV)	8.69	60.5	16.4	57.2
$\Delta E_{ST(CA10)}^{PS}$ (meV)	8.56	58.4	16.3	56.2
$\Delta E_{ST(CA15)}^{PS}$ (meV)	8.51	57.9	16.1	55.7
$\Delta E_{ST(CA20)}^{PS}$ (meV)	8.46	57.2	16.0	55.4
$\Delta E_{ST(CA25)}^{PS}$ (meV)	8.35	56.8	15.9	55.0
$\Delta E_{ST(CA0)}^{PMMA}$ (meV)	8.74	59.5	16.5	57.0
$\Delta E_{ST(CA5)}^{PMMA}$ (meV)	8.63	58.7	16.4	56.4
$\Delta E_{ST(CA10)}^{PMMA}$ (meV)	8.56	57.9	16.3	55.6
$\Delta E_{ST(CA15)}^{PMMA}$ (meV)	8.47	57.2	16.1	55.3
$\Delta E_{ST(CA20)}^{PMMA}$ (meV)	8.44	56.7	16.0	55.1
$\Delta E_{ST(CA25)}^{PMMA}$ (meV)	8.34	56.4	15.9	54.3

S11. Prompt lifetime on TCSPC method and fitting value.

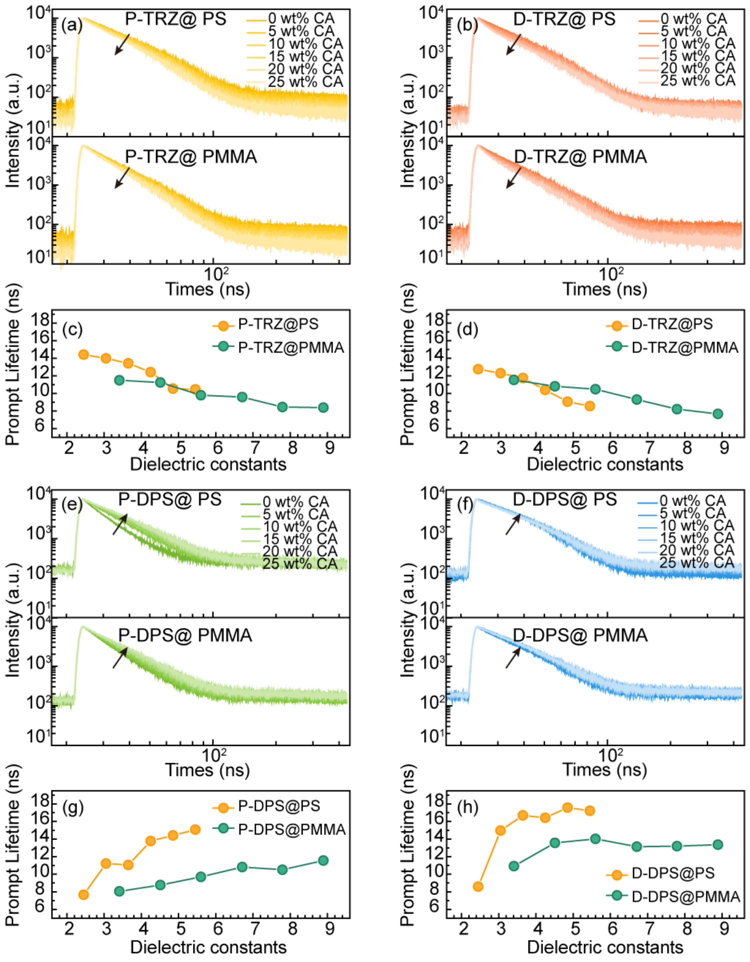

Figure S6. TCSPC measurement of prompt fluorescence and a tendency of τ_{PF} for P-TRZ (a,c), D-TRZ (b,d), P-DPS (e,g), and D-DPS (f,h) in PS and PMMA films with increasing CA concentration.

S12. Vibrational modes.

Table S11. The dominant modes displayed in Huang–Rhys.

	vibrational mode	vibrational frequency (cm^{-1})
P-TRZ	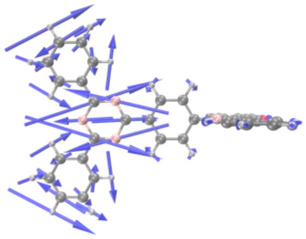	1593.36
D-TRZ	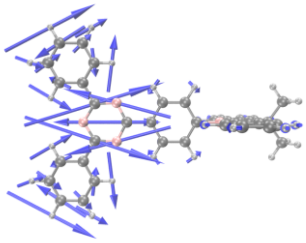	1593.45
P-DPS	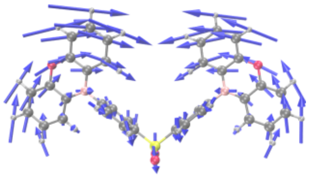	7.94
D-DPS	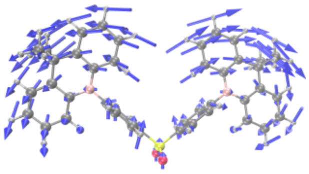	6.73
